# Increased glucosylceramide production leads to decreased cell energy metabolism and lowered tumor marker expression in non-cancerous liver cells

**DOI:** 10.1007/s00018-021-03958-9

**Published:** 2021-10-09

**Authors:** Marthe-Susanna Wegner, Nina Schömel, Ellen M. Olzomer, Sandra Trautmann, Catherine Olesch, Frances L. Byrne, Bernhard Brüne, Robert Gurke, Nerea Ferreirós, Andreas Weigert, Gerd Geisslinger, Kyle L. Hoehn

**Affiliations:** 1 Pharmazentrum Frankfurt/ZAFES, Institute of Clinical Pharmacology, Johann Wolfgang Goethe University, House 74, Theodor Stern-Kai 7, 60590 Frankfurt am Main, Germany; 2 School of Biotechnology and Biomolecular Sciences, University of New South Wales, Sydney, NSW 2052 Australia; 3 Faculty of Medicine, Institute of Biochemistry I, Johann Wolfgang Goethe University, Theodor Stern-Kai 7, 60590 Frankfurt am Main, Germany; 4 Fraunhofer Institute for Translational Medicine and Pharmacology (ITMP), Theodor Stern-Kai 7, 60590 Frankfurt am Main, Germany

**Keywords:** Glycolysis, Oxidative phosphorylation, Mitochondrial ROS, HCC marker, GEMs

## Abstract

Hepatocellular carcinoma (HCC) is one of the most difficult cancer types to treat. Liver cancer is often diagnosed at late stages and therapeutic treatment is frequently accompanied by development of multidrug resistance. This leads to poor outcomes for cancer patients. Understanding the fundamental molecular mechanisms leading to liver cancer development is crucial for developing new therapeutic approaches, which are more efficient in treating cancer. Mice with a liver specific UDP-glucose ceramide glucosyltransferase (UGCG) knockout (KO) show delayed diethylnitrosamine (DEN)-induced liver tumor growth. Accordingly, the rationale for our study was to determine whether UGCG overexpression is sufficient to drive cancer phenotypes in liver cells. We investigated the effect of UGCG overexpression (OE) on normal murine liver (NMuLi) cells. Increased UGCG expression results in decreased mitochondrial respiration and glycolysis, which is reversible by treatment with EtDO-P4, an UGCG inhibitor. Furthermore, tumor markers such as FGF21 and EPCAM are lowered following UGCG OE, which could be related to glucosylceramide (GlcCer) and lactosylceramide (LacCer) accumulation in glycosphingolipid-enriched microdomains (GEMs) and subsequently altered signaling protein phosphorylation. These cellular processes lead to decreased proliferation in NMuLi/UGCG OE cells. Our data show that increased UGCG expression itself does not induce pro-cancerous processes in normal liver cells, which indicates that increased GlcCer expression leads to different outcomes in different cancer types.

**Graphic abstract:**

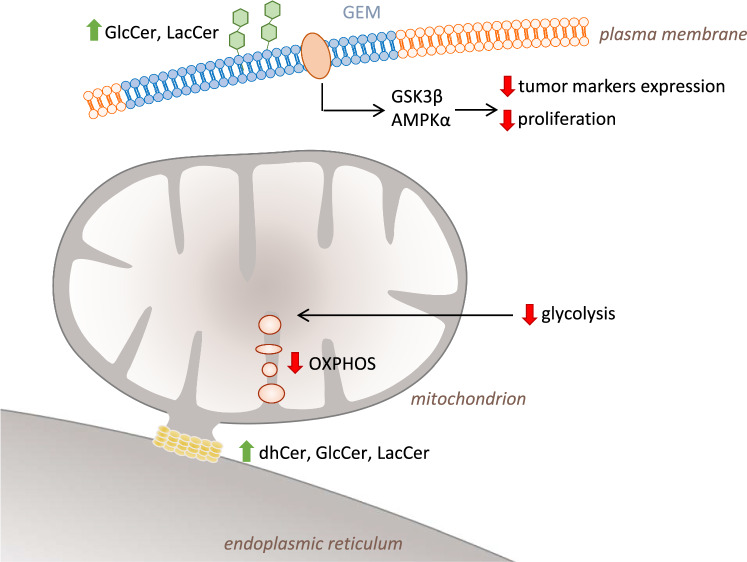

**Supplementary Information:**

The online version contains supplementary material available at 10.1007/s00018-021-03958-9.

## Introduction

The most frequent type of primary liver cancer is hepatocellular carcinoma (HCC), which is characterized by transformation of liver cells into tumor cells. Only 30% of HCC patients are suitable for liver resection or transplantation leading to a high death rate for the patients. In addition, development of multidrug resistance during pharmacotherapy is common in HCC patients [42]. In recent years, progress has been made regarding pharmacologic treatment of HCC. The multikinase inhibitor sorafenib is a first-line therapy for advanced HCC (reviewed in [13]). Unfortunately, sorafenib is effective only in the minority of HCC patients and increases life span only by roughly 12 weeks [2, 25]. In 2018, lenvatinib (also a multikinase inhibitor) was approved from the Food and Drug Administration (FDA) as a first-line treatment of patients with unresectable HCC (reviewed in [13]). Despite exhibiting a higher response rate of lenvatinib over sorafenib the REFLECT trial showed no inferiority of one of the drugs (reviewed in [32]). On May 29, 2020, the FDA approved programmed cell death protein 1 (PD1)/programmed cell death 1 ligand 1 (PD-L1) immune checkpoint inhibitor atezolizumab in combination with angiogenesis growth factor (VEGF) inhibitor bevacizumab for patients with unresectable or metastatic HCC without prior systemic therapy. Combination therapy with atezolizumab and bevacizumab provides superior outcome for the patients compared with sorafenib, but the incidence of serious adverse events is increased (reviewed in [32]). Despite these novel therapeutic approaches, for many of the HCC patients only palliative care applies. This reveals the importance of investigating the molecular mechanisms of HCC development. Furthermore, understanding these basic mechanisms contributes to development of new therapeutic strategies for prevention and treatment of HCC. UDP-glucose ceramide glucosyltransferase (UGCG) gene expression is altered in 0.8% of HCC patients (TCGA, Firehose Legacy) [9, 14]. UGCG overexpression is linked to multidrug resistance development in cancer cells (reviewed in [45]), which is ascribed to the unique ability of UGCG to transfer UDP-glucose to ceramide, which results in de novo synthesis of glucosylceramide (GlcCer, cerebroside). Since GlcCer is the precursor for all complex glycosphingolipids (GSLs), UGCG is the key enzyme of GSL metabolism. Synthesized in the Golgi apparatus, GSLs are essential for cellular survival. They are also involved in signal transmission processes. By adding a galactose molecule to GlcCer, lactosylceramide (LacCer, globoside) is produced. The more complex GSLs globosides (e.g. globotriaosylceramide (Gb3)) and gangliosides (e.g. monosialodihexosylganglioside (GM3)) are synthesized by adding monosaccharides to LacCer (reviewed in [16]).

The lysosomal storage disease Morbus Gaucher is characterized by a mutation of the glucocerebrosidase (GBA) gene, which results in GlcCer accumulation. Morbus Gaucher patients exhibit hypermetabolism [11]. In addition, these patients show an increased risk for liver cancer [33]. Hence, it is interesting to identify the role of GlcCer in liver cell metabolism and whether GlcCer contributes to the pathology of liver tumors.

Currently, only two studies show the involvement of UGCG in the molecular processes of HCC. Jennemann et al. showed increased UGCG expression in HCC tissue compared to non-cancerous tissue [21]. Diethylnitrosamine (DEN)-induced liver tumors in mice, which exhibit a liver specific UGCG knockout (KO), show delayed tumor growth. This is ascribable to decelerated cytokinesis, whereas, the authors do not mention which signaling pathway is involved in the described effects. Furthermore, the ganglioside GM2 is hardly detectable, whereas, the sphingomyelin concentration is increased. Possibly, this is a cellular mechanism to avoid ceramide induced apoptosis [21]. This study was unable to show that a lack of GSLs protects from liver tumor development, which implicates that beside UGCG, other proteins/pathways play a role in HCC development. The second study was also carried out with liver cancer cells and showed sorafenib induced UGCG expression resulting in sorafenib resistance [40]. Following UGCG inhibition, no alterations of phosphatidylinositol 3-kinase (PI3K)/protein kinase B (AKT) and rapidly accelerated fibrosarcoma (RAF)/ mitogen-activated protein kinase (MAPK)/extracellular signal-regulated kinase (ERK) signaling could be detected in liver cancer cells [33]. One reason for this could be that tumor cells were used in these studies in which the described signaling pathways are rather induced in the onset of carcinogenesis.

To investigate early onset of UDP-glucose ceramide glucosyltransferase (UGCG)-mediated pro-cancerous changes in normal liver cells, we overexpressed UGCG in NMuLi cells, analyzed several key cellular processes and measured the expression of tumor markers. Following UGCG overexpression (OE), NMuLi cells show decreased mitochondrial respiration and glycolysis accompanied by increased mitochondrial superoxide levels and decreased tumor marker expression (graphical abstract). We also detected increased dihydroceramide (dhCer) levels in endoplasmic reticulum (ER)/mitochondria fractions in NMuLi/UGCG OE cells. Furthermore, altered signaling of glycogen synthase kinase 3 β (GSK3β), adenosine monophosphate (AMP)-activated protein kinase (AMPKα) and 3′-phosphoinositide-dependent kinase 1 (PDK1) was detected, presumably based on glucosylceramide (GlcCer) and lactosylceramide (LacCer) accumulation in glycosphingolipid-enriched microdomains (GEMs) resulting in decreased cell proliferation.

## Results

### UGCG OE verification

**Fig. 1:**
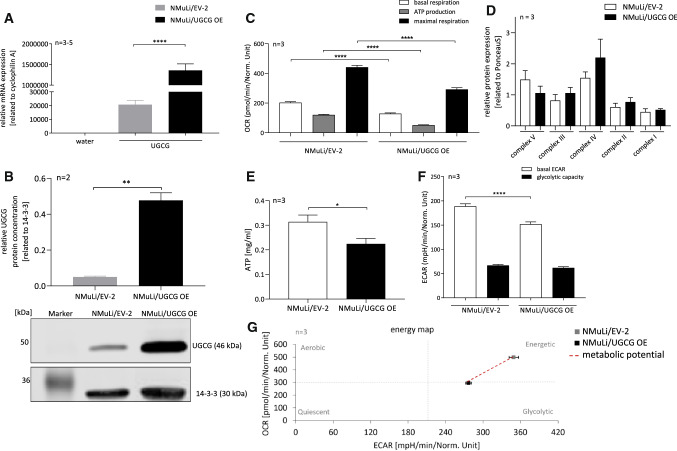
Stable UGCG OE in NMuLi cells and oxidative phosphorylation (OXPHOS) and glycolysis of NMuLi/UGCG OE and control cells. **A** Expression analysis of UGCG mRNA by qRT-PCR. mRNA expression is normalized to the housekeeping gene cyclophilin A. Data are represented as a mean of *n* = 3 − 5 ± SEM. Unpaired *t* test with Welch’s correction. **B** Upper part: Densitometrical analysis of UGCG protein concentration by Western blot analysis. Lower part: representative blot. Data are normalized to the housekeeper 14-3-3 and represented as a mean of *n* = 2 ± SEM. Unpaired *t* test with Welch’s correction. **C** Oxygen consumption rate (OCR) determined by Seahorse XFe analyzer. ATP production (basal respiration—respiration after oligomycin injection) and maximal respiration rate (respiration after N5,N6-bis(2-fluorophenyl)-[1,2,5]oxadiazolo[3,4-b]pyrazine-5,6-diamine (BAM15) injection—respiration after antimycin a/rotenone injection). Data are presented as mean of *n* = 3 ± SEM. Tukey’s multiple comparisons test. **D** Densitometrical analysis of OXPHOS complexes I–V protein concentrations by Western blot analysis. Protein expression is normalized to Ponceau dye. Data are presented as a mean of *n* = 3 ± SEM. Unpaired *t* test with Welch’s correction. **E** ATP concentration determination by a luciferase-based bioluminescent assay. Data are presented as a mean of *n* = 3 ± SEM. Unpaired *t* test with Welch’s correction. **F** Extracellular acidification rate (ECAR) quantified by Seahorse XFe analyzer. Glycolytic capacity is deduced from ECAR after oligomycin treatment—basal ECAR. Data are presented as mean of *n* = 3 ± SEM. Tukey’s multiple comparisons test. **G** Energy map of NMuLi/EV-2 and NMuLi/UGCG OE cells. *p ≤ 0.05, **p ≤ 0.01, ****p ≤ 0.0001

We performed lentiviral transduction to overexpress UDP-glucose ceramide glucosyltransferase (UGCG) in non-cancerous murine liver cells (NMuLi/UGCG OE cells). Overexpression (OE) of UGCG is confirmed via mRNA (Fig. 1A) and protein (Fig. 1B, S1A) level analysis compared to control cells (NMuLi/EV-2 cells).

### UGCG OE decreases glycolysis and OXPHOS

**Fig. 2:**
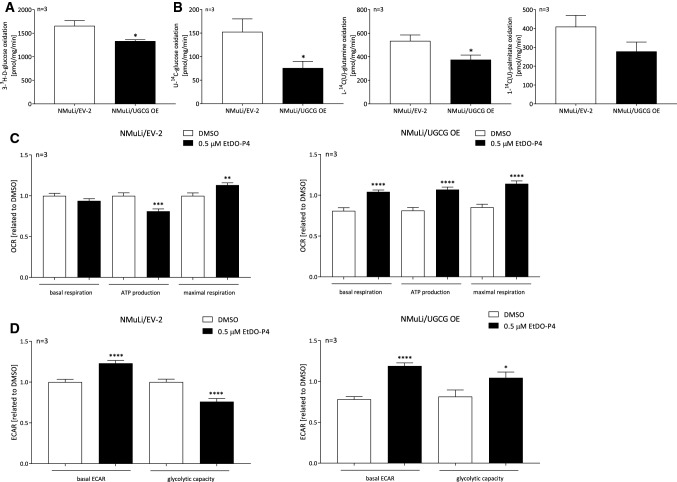
Glycolysis and substrate oxidation in NMuLi cells and oxygen consumption rate (OCR) and extracellular acidification rate (ECAR) following EtDO-P4 treatment of NMuLi/UGCG OE and control cells. **A** Using 3-^3^H-D-glucose and subsequently measuring released labeled H_2_O, anaerobic glycolysis was determined. Data are normalized to protein concentration and presented as mean of *n* = 3 ± SEM. Unpaired *t* test with Welch’s correction. **B** Substrate oxidation was analyzed by evolved ^14^CO_2_ following U-^14^C-glucose, L-^14^C-glutamine, and 1-^14^C-palmitate treatment. Data are normalized to protein concentration and presented as mean of n = 3 ± SEM. Unpaired *t* test with Welch’s correction. **C** Oxygen consumption rate (OCR) following EtDO-P4 treatment determined by Seahorse XFe analyzer. ATP production (basal respiration—respiration after oligomycin injection) and maximal respiration rate (respiration after N5,N6-bis(2-fluorophenyl)-[1,2,5]oxadiazolo[3,4-b]pyrazine-5,6-diamine (BAM15) injection—respiration after antimycin a/rotenone injection). Data are presented as mean of *n* = 3 ± SEM. Tukey’s multiple comparisons test. **D** Extracellular acidification rate (ECAR) following EtDO-P4 treatment quantified by Seahorse XFe analyzer. Glycolytic capacity is deduced from ECAR after oligomycin treatment—basal ECAR. Data are presented as mean of *n* = 3 ± SEM. Tukey’s multiple comparisons test. *p ≤ 0.05, **p ≤ 0.01, ***p ≤ 0.001, ****p ≤ 0.0001

First we investigated the effect of an UGCG OE on liver cell metabolism. Basal mitochondrial respiration is significantly decreased in NMuLi/UGCG OE cells compared to control cells (Fig. 1C, S1B). Adenosine triphosphate (ATP) production (basal respiration —respiration after oligomycin injection) and maximal respiration rate (respiration after N5,N6-bis(2-fluorophenyl)-[1,2,5]oxadiazolo[3,4-b]pyrazine-5,6-diamine (BAM15) injection —respiration after antimycin a/rotenone injection) are also significantly decreased in NMuLi/UGCG OE cells compared to control cells (Fig. 1C, S1B). No statistically significant differences of mitochondrial oxidative phosphorylation (OXPHOS) complex protein concentrations between NMuLi/UGCG OE and control cells have been detected (Fig. 1D, S1C). However, ATP levels are decreased in UGCG overexpressing cells (Fig. 1E). Furthermore, the basal glycolytic rate (basal extracellular acidification rate (ECAR)) is lowered in NMuLi/UGCG OE cells (Fig. 1F, S1D). Interestingly, glycolytic capacity is not significantly changed between the cells (Fig. 1F). The energy map shows that following UGCG OE, NMuLi cells transform from an energetic to a glycolytic/quiescence state (Fig. 1G). We analyzed glycolytic (J_ATPglc_) and oxidative ATP (J_ATPox_) production rates from raw extracellular acidification and respiration data, which confirm the metabolic shift to a glycolytic/quiescence state following UGCG OE (S1E). NMuLi/UGCG knockdown (KD) cell data (verified KD in S1F) confirm these findings by showing increased basal respiration, ATP production, maximal respiration (S1G) and glycolytic capacity in NMuLi/UGCG KD cells (S1H) compared to control cells. Lowered glycolysis in NMuLi/UGCG OE cells is also shown by decreased evolved ^3^H_2_O concentration following supplementing 3-^3^H-D-glucose to the media (Fig. 2A). Decreased oxidative metabolism is verified by substrate competition assay data with [U-^14^C]-labeled glucose, [L-^14^C(U)]-labeled glutamine and [1-^14^C(U)]-labeled palmitate (Fig. 2B). OE of UGCG lowers levels of captured ^14^CO_2_, which demonstrates that UGCG OE decreases substrate oxidation in NMuLi cells (Fig. 2B). Interestingly, basal respiration in NMuLi/UGCG OE cells can be rescued by treatment with 0.5 μM EtDO-P4, an UGCG inhibitor, whereas, no EtDO-P4 effect on basal respiration in control cells was detected (Fig. 2C). ATP production of NMuLi/UGCG OE cells is 0.4-fold increased following treatment with EtDO-P4, whereas, ATP production in NMuLi/EV-2 cells is lowered (Fig. 2C). EtDO-P4 also improves maximal respiration of NMuLi/EV-2 cells, but to a smaller extent when compared to NMuLi/UGCG OE cells (Fig. 2C). Furthermore, basal ECAR is 0.25-fold higher in NMuLi/EV-2 cells and 0.5-fold higher in NMuLi/UGCG OE cells following EtDO-P4 stimulation (Fig. 2D). Glycolytic capacity is improved in NMuLi/UGCG OE cells by 0.5 μM EtDO-P4 stimulation, whereas, in control cells EtDO-P4 leads to lowered glycolytic capacity (Fig. 2D). The data are in line with KD data, since EtDO-P4 has no effect on the OCR and ECAR in NMuLi/UGCG KD cells (S1H).

### UGCG does not affect mitochondrial mass, but mitochondrial ROS levels

**Fig. 3:**
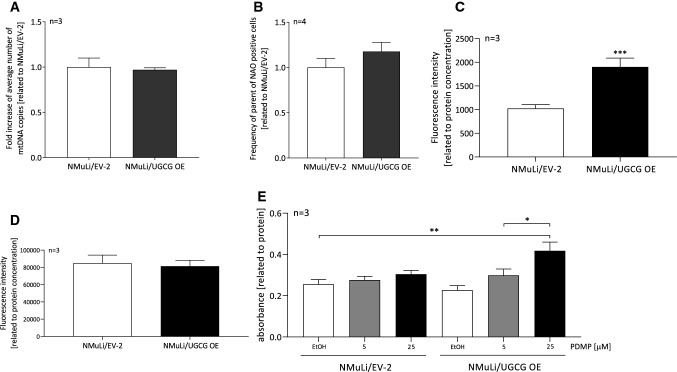
Mitochondrial mass and cellular stress determination in NMuLi/UGCG OE and cells. **A** Mitochondrial DNA (mtDNA) copy number was quantified by using the NovaQUANT Human Mitochondrial to Nuclear DNA Ratio Kit. Data are presented as a mean of *n* = 3 ± SEM. Unpaired *t* test with Welch’s correction. **B** Mitochondrial mass determined by using nonyl acridine orange (NAO) in flow cytometry. Data are presented as a mean of *n* = 4 ± SEM. Unpaired *t* test with Welch’s correction. **C** Mitochondrial reactive oxygen species (ROS) (superoxide) levels were analyzed using the fluorogenic reagent MitoSOX. Data are normalized to protein concentration and presented as a mean of *n* = 3 ± SEM. Unpaired *t* test with Welch’s correction. **D** Total ROS levels were analyzed by a fluorescence-based assay. Data are normalized to protein concentration and presented as mean of *n* = 3 ± SEM. Unpaired *t* test with Welch’s correction. **E** 3-(4,5-dimethylthiazol-2-yl)-2,5-diphenyltetrazolium Bromide (MTT) assay following d,l-threo-1-phenyl-2-decanoylamino-3-morpholino-1-propanol (PDMP) treatment. Data are normalized to protein concentration and are presented as a mean of *n* = 3 ± SEM. Sidak’s multiple comparisons test. *p ≤ 0.05, **p ≤ 0.01, ***p ≤ 0.001

To exclude the possibility that UGCG-dependent effects on cell metabolism are related to changed mitochondrial mass, we determined the number of mitochondrial DNA (mtDNA) copies. No statistically significant differences between the mtDNA in NMuLi/UGCG OE and control cells were detected (Fig. 3A). In addition, we used the fluorescent mitochondrial dye nonyl acridine orange (NAO) for mitochondrial mass analysis. Following cell labeling with NAO, we analyzed vital mitochondria by flow cytometry. No significant differences between NMuLi/UGCG OE and control cells were detected (Fig. 3B). Since mitochondrial respiration is clearly UGCG-dependently regulated, we determined mitochondrial superoxide in NMuLi cells. Mitochondrial superoxide increases in NMuLi cells in an UGCG-dependent manner when compared to the control (Fig. 3C), whereas, the total reactive oxygen species (ROS) level is unchanged (Fig. 3D). No significant changes in nicotinamide adenine dinucleotide (NAD) + , NAD + hydrogen (NADH) concentrations (S2A) and antioxidant capacity (S2B) between NMuLi/UGCG OE and control cells were detected. 3-(4,5-dimethylthiazol-2-yl)-2,5-diphenyltetrazolium bromide (MTT) assay data show increased viability of NMuLi/UGCG OE cells following 25 μM d,l-threo-1-phenyl-2-decanoylamino-3-morpholino-1-propanol (PDMP), an UGCG inhibitor, treatment compared to mock control of NMuLi/EV-2 cells (Fig. 3E). In summary, UGCG-dependent effects on cell metabolism are not related to mitochondrial mass change, mitochondrial superoxide is increased following UGCG OE and NMuLi/UGCG OE cell viability is increased by inhibiting UGCG.

### UGCG-derived glycosphingolipids change sphingolipid composition of ER/mitochondria fractions

**Fig. 4:**
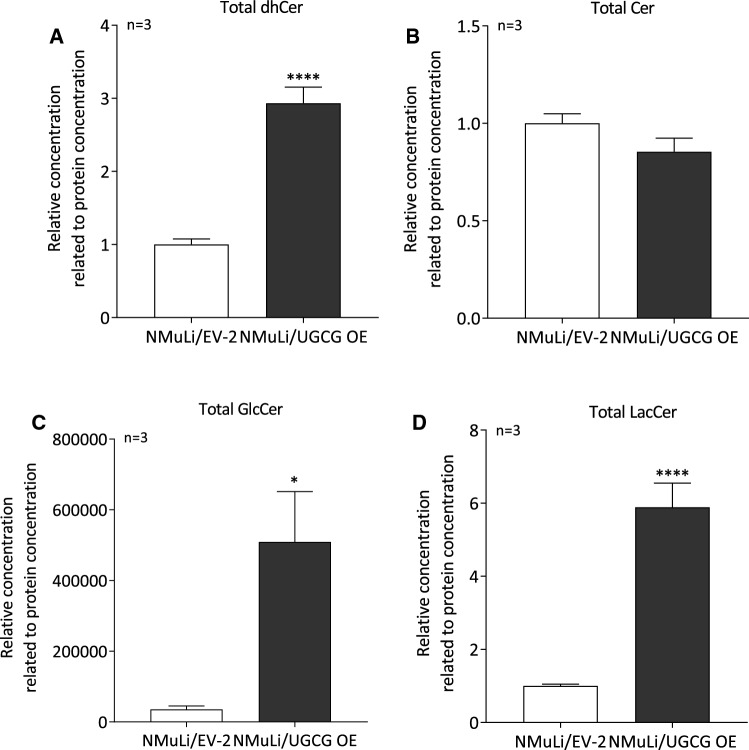
Determination of total dihydrocermide (dhCer), ceramide (Cer), glucosylceramide (GlcCer) and lactosylceramide (LacCer) content in endoplasmic reticulum (ER)/mitochondria fractions of NMuLi/UGCG OE and control cells by LC–MS/MS. Following isolation of ER/mitochondria fractions, total **A** dihydroceramide (dhCer) (C_16:0_-, C_18:0_-, C_24:0_-, C_24:1_-dhCer), **B** ceramide (Cer) (C_14:0_-, C_16:0_-, C_18:0_-, C_20:0_-, C_22:0_-, C_24:0_-, C_24:1_-Cer) **C** glucosylceramide (GlcCer) (C_16:0_-, C_18:0_-, C_24:1_-GlcCer) and **D** lactosylceramide (LacCer) (C_16:0_-, C_18:0_-, C_24:0_-, C_24:1_-LacCer) levels were determined by LC–MS/MS. Data are represented as a mean of *n* = 3 ± SEM. Unpaired *t* test with Welch’s correction. *p ≤ 0.05, ****p ≤ 0.0001

Endoplasmic reticulum (ER) and mitochondria are closely connected by mitochondria associated ER membranes (MAMs) [35]. UGCG OE clearly affects mitochondrial respiration and therefore, we analyzed total dihydroceramide (dhCer), ceramide (Cer), glucosylceramide (GlcCer) and lactosylceramide (LacCer) levels in ER/mitochondria fractions by LC–MS/MS. The data reveal significantly increased total dhCer (Fig. 4A), GlcCer (Fig. 4C) and LacCer levels (Fig. 4D) in NMuLi/UGCG OE cells compared to the control. Total Cer levels (Fig. 4B) are unchanged following UGCG OE. UGCG KD data verify this finding by showing significantly decreased total GlcCer and LacCer levels (S2C), whereas, total dhCer levels are unchanged and total Cer levels are increased in NMuLi/UGCG KD cells compared to control cells (S2C).

### UGCG-derived glycosphingolipids accumulate in GEMs and change cell signaling pathways

**Fig. 5:**
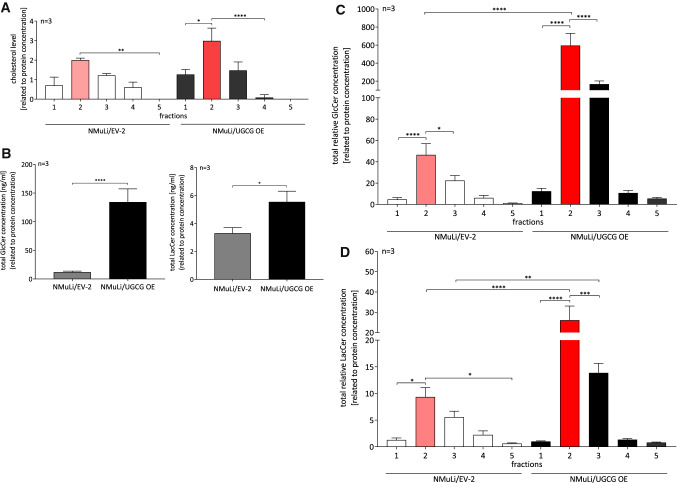
Glycosphingolipid-enriched microdomains (GEMs) verification and glucosylceramide (GlcCer) and lactosylceramide (LacCer) content of GEMs in NMuLi/UGCG OE and control cells. **A** GEMs were isolated by sucrose density centrifugation and cholesterol-level determination by ELISA of fraction 1–5 was performed. Data are represented as a mean of *n* = 3 ± SEM. Tukey’s multiple comparisons test. **B** GEMs were isolated and total glucosylceramide (GlcCer) (C_16:0_-, C_18:0_-, C_24:1_-GlcCer) and lactosylceramide (LacCer) (C_16:0_-, C_18:0_-, C_24:0_-, C_24:1_-LacCer) levels of fractions 1–8 were determined by LC–MS/MS. Results of fraction 1–8 are summarized. Data are represented as a mean of *n* = 3 ± SEM. Unpaired *t* test with Welch’s correction. **C** GEMs were isolated and total GlcCer (C_16:0_-, C_18:0_-, C_24:1_-GlcCer) levels of fractions 1–5 were determined by LC–MS/MS. Data are represented as a mean of *n* = 3 ± SEM. Tukey’s multiple comparisons test. **D** GEMs were isolated and total LacCer (C_16:0_-, C_18:0_-, C_24:0_-, C_24:1_-LacCer) levels of fractions 1–5 were determined by LC–MS/MS. Data are represented as a mean of *n* = 3 ± SEM. Tukey’s multiple comparisons test. *p ≤ 0.05, **p ≤ 0.01, ***p ≤ 0.001, ****p ≤ 0.0001

**Fig. 6:**
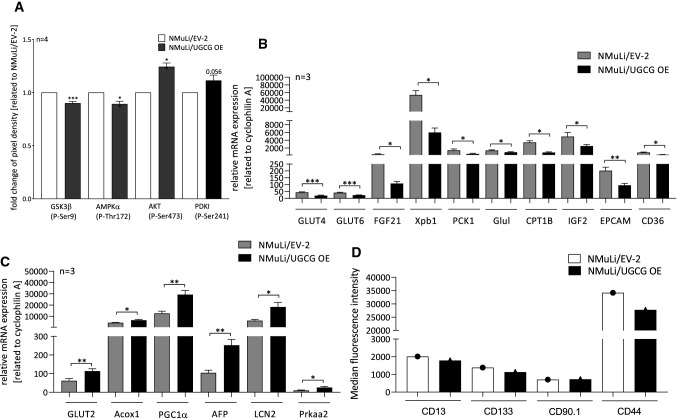
Protein phosphorylation status and mRNA expression analysis of tumor marker in NMuLi/UGCG and control cells by qRT-PCR. **A** Phosphorylation status of GSK3β, AMPKα, AKT and PDKI determined by an antibody array. Data are represented as a mean of *n* = 4 ± SEM. Unpaired *t* test with Welch’s correction. **B** Reduced mRNA levels following UGCG OE. The mRNA expression is related to the housekeeping gene cyclophilin A. Data are represented as a mean of *n* = 3 ± SEM. Unpaired *t* test with Welch’s correction. **C** Induced mRNA levels following UGCG OE. The mRNA expression is related to the housekeeping gene cyclophilin A. Data are represented as a mean of *n* = 3 ± SEM. Unpaired *t* test with Welch’s correction. **D** Detection of CD13^+^, CD133^+^, CD90.1^+^ and CD44^+^ cells by flow cytometry. *p ≤ 0.05, **p ≤ 0.01, ***p ≤ 0.001

To investigate whether UGCG-derived glycosphingolipids (GSL) arrive at glycosphingolipid-enriched microdomains (GEMs), we analyzed GSL levels in GEMs by LC–MS/MS. GEMs fractions were obtained by saccharose density gradient centrifugation as described in Wegner et al. [46] and fraction 2 (out of 8) is verified as the GEM containing fraction by determination of cholesterol level in NMuLi/UGCG OE and control cells (Fig. 5A). Previously, we identified fraction 2 and/or 3 as GEMs containing fractions in breast cancer cells [37, 46]. Accordingly, fractions 1–5 were analyzed. In addition, our LC–MS/MS data show the highest concentration of GlcCer and LacCer in fraction 2 (out of 8 fractions) for NMuLi/EV-2 and fraction 2 and 3 (out of 8 fractions) for NMuLi/UGCG OE cells (data not shown). Our LC–MS/MS data reveals an increase of total GlcCer (fraction 1 to 8) and subsequently total LacCer (GlcCer are precursor for LacCer) concentrations in NMuLi/UGCG OE cells (Fig. 5B), which verifies UGCG OE in NMuLi cells. Furthermore, UGCG-derived GlcCer and LacCer, mainly accumulate in fraction 2 and 3 (Fig. 5C, D). Notably, NMuLi/UGCG OE cells exhibit a 12-fold increase of GlcCer concentration in fraction 2 and a sixfold increase in fraction 3 compared to control cells (Fig. 5C). LacCer levels are increased 2.5-fold in fraction 2 and 3 of NMuLi/UGCG OE cells compared to control cells (Fig. 5D). Accordingly, UGCG OE leads to sphingolipid composition changes of GEMs or to increased numbers of GEMs. Since GEMs are signaling platforms for cellular processes and proteins residing in GEMs are influenced by lipid composition, we analyzed phosphorylation of key signaling proteins. UGCG OE leads to a statistically significant decrease of phosphorylated GSK3β (P-Ser9) and AMPKα (P-Thr172) and an increase of AKT (P-Ser473) and PDKI (P-Ser241), whereas, the latter is not significant (Fig. 6A, S2D). We confirmed decreased phosphorylated GSK3β (P-Ser9), AMPKα (P-Thr172) and increased phosphorylated PDKI (P-Ser241) by Western Blot analysis (S2E, S2F). Contradictory, phosphorylated AKT (P-Ser473) was decreased when determined by Western Blot analysis (S2E, S2F).

### UGCG OE decreases tumor marker expression

To get insights about the influence of UGCG OE on malignant transformation of liver cells, mRNA expression of selected tumor cell markers was analyzed by quantitative real-time-PCR (qRT-PCR). Following OE of UGCG, mRNA concentrations of the tumor markers glucose transporter type 4 (GLUT4), glucose transporter type 6 (GLUT6), fibroblast growth factor 21 (FGF21), X-box binding protein 1 (Xpb1), phosphoenolpyruvate carboxykinase 1 (PCK1), glutamate-ammonia ligase (Glul), carnitine palmitoyltransferase 1B (CPT1B), insulin-like growth factor 2 (IGF2), epithelial cell adhesion molecule (EPCAM) and cluster of differentiation 36 (CD36) are significantly decreased in NMuLi/UGCG OE cells (Fig. 6B). Interestingly, we detected increased mRNA levels of glucose transporter type 2 (GLUT2), peroxisomal acyl-coenzyme A oxidase 1 (Acox1), peroxisome proliferator-activated receptor γ coactivator 1α (PGC1α), alpha fetoprotein (AFP), lipocalin-2 (LCN2) and protein kinase AMP-activated catalytic subunit α 2 (Prkaa2) (Fig. 6C). GLUT2, Acox1, PGC1α, AFP, LCN2, CD36 and Prkaa2 mRNA levels are decreased in NMuLi/UGCG KD cells (S2G). In addition, we analyzed protein expression of selected liver cancer stem cell markers by flow cytometry. Following UGCG OE, protein expression of CD13^+^, CD133^+^ and CD44^+^ is reduced on liver cells, whereas, CD90.1^+^ expression is unchanged (Fig. 6D). In summary, these data reveal that UGCG OE reduces expression of tumor cell markers on normal liver cells.

### UGCG OE lowers normal liver cell proliferation

**Fig. 7:**
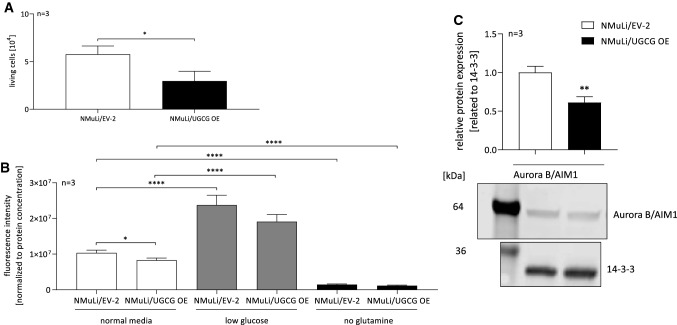
Cell proliferation of NMuLi/UGCG OE and control cells. **A** Living cell number determination by counting with a Neubauer counting chamber under normal media conditions. Data are represented as a mean of *n* = 3 ± SEM. Unpaired *t* test with Welch’s correction. **B** Cell number determination by CyQUANT^®^ NF Cell Proliferation Assay under normal media, low glucose and no glutamine conditions. Data are represented as a mean of *n* = 3 ± SEM. Tukey’s multiple comparisons test. **C** Upper part: Densitometrical analysis of Aurora B/AIM1 protein concentration by Western blot analysis. Lower part: representative blot. Data are normalized to the housekeeper pan 14-3-3 and represented as a mean of *n* = 3 ± SEM. Unpaired *t* test with Welch’s correction. *p ≤ 0.05, ****p ≤ 0.0001

Since UGCG OE in normal liver cells leads to decreased OXPHOS and glycolysis, and cell viability can be improved by treatment with an UGCG inhibitor, the direct influence of UGCG OE on cell proliferation was investigated. NMuLi/UGCG OE cells proliferate significantly less than control cells (Fig. 7A). We verified the data by performing a CyQUANT^®^ NF Cell Proliferation Assay, which is based on a cell-permeant binding of the dye to DNA and therefore can be used for cell number determination. We could verify less NMuLi/UGCG OE proliferation compared to control cells under normal media conditions (Fig. 7B). Interestingly, low glucose media conditions increase cell numbers for both NMuLi/UGCG OE and control cells (Fig. 7B). Glutamine depletion decreases NMuLi/UGCG OE and control cell proliferation (Fig. 7B) indicating that glutamine dependency is not given following UGCG OE. Interestingly, Aurora B/AIM1 protein concentration is significantly decreased following UGCG overexpression in NMuLi cells (Fig. 7C, S2H). Aurora B/AIM1 is essential for proper cytokinesis.

## Materials and methods

### Cell culture

The normal murine liver cell line NMuLi (American Type Culture Collection (ATCC) CRL-1638, Manassas, Virginia, US) was cultured in Dulbecco’s Modified Eagle Medium (DMEM) containing high glucose, L-glutamine, sodium bicarbonate, 10% fetal bovine serum (FBS) (Sigma Aldrich, St. Louis, Missouri, US) and 1% Penicillin/Streptomycin. Cells were cultured at 37 °C in an atmosphere containing 5% CO_2_. 1 μg/ml puromycin (Sigma Aldrich, St. Louis, Missouri, US) was added for selection of stably transfected NMuLi/EV-2 and NMuLi/UGCG OE cells.

### Cloning of UGCG and subsequent lentiviral transduction

Primers were designed with BamHI (5’) and Sal I (3’) restriction enzymes sites at the ends. UGCG was amplified by PCR and the PCR product digested and purified. Subsequently, the PCR product was ligated into purified pBABE-puro that was digested with BamHI and Sal I. As a control the pBABE-puro (Empty Vector (EV)-2) was used (NMuLi/EV-2 cells). The pBABE-puro-UGCG construct was then amplified and transfected into Platinum-E cells. Retrovirus-containing supernatant was collected 3 days later and used to infect NMuLi cells. For viral transduction, 8 × 10^4^ NMuLi cells were seeded in a 6-well. 1 ml DMEM with 10% FBS and no antibiotics was added. NMuLi cells were incubated with 4 µg/ml polybrene and 1/10 viral supernatant for 48 h at 37 °C. Subsequently, the viral supernatant was removed, and stable cell lines were selected by puromycin treatment (1 µg/ml).

### qRT-PCR

Quantitative real-time PCR (qRT-PCR) was performed as described previously [37]. Briefly, the RNeasy Mini Kit (QIAGEN, Hilden, Germany) was used to isolate total RNA. 1000 ng RNA were applied to synthesize cDNA using the Verso cDNA Synthesis Kit (Thermo Fisher Scientific, Waltham, USA). Gene-specific PCR products were quantified utilizing Fast SYBR™ Green Master Mix (Thermo Fisher Scientific, Waltham, USA) on a QuantStudio 5 Real-time PCR System (Thermo Fisher Scientific, Waltham, USA). Relative mRNA expression was calculated according to the ΔΔCt method. Relative values were normalized to cyclophilin A (or glyceraldehyde-3-phosphate dehydrogenase (GAPDH) for NMuLi/UGCG KD and control). All primers (except GAPDH), as listed in Table 1, were purchased from Sigma Aldrich (St Louis, Missouri, US). GAPDH primers were purchased from Biomers (Ulm, Germany).

**Table 1:** qRT-PCR primer sequences

Gene	Forward primer sequence (5′ → 3′)	Reverse primer sequence (5′ → 3′)
Cyclophilin A	cgatgacgagcccttgg	tctgctgtctttggaactttgtc
GAPDH	aggtcggtgtgatttg	Tgtagaccataggtc
UGCG	ggaatggccttgttcggct	Cggctgtttgtctgttgcc
GLUT4	gtaacttcattgtcggcatgg	agctgagatctggtcaaacg
GLUT6	tgtgtcagcatccgtcatgtt	tagttgaaggctgtgtcccc
FGF21	atggaatggatgagatctagagttgg	tcttggtggtcatctgtgtagagg
Xpb1	ggccttgtggttgagaaccaggag	gaatgcccaaaaggatatcagactc
PCK1	tgacaactgttggctggctc	Gttttggggatgggcactg
Glul	gttcccacttgaacaaaggca	acccagatatacatggcttgga
CPT1B	ggaccgtgaagagatcaagc	ctgggatgcgtgtagtgttg
IGF2	gttggctgaatatgggacagtg	gtagtcctcaaactgatgccc
EPCAM	gcggctcagagagactgtg	ccaagcatttagacgccagttt
CD36	gatgacgtggcaaagaacag	tcctcggggtcctgagttat
GLUT2	tgtgctgctggataaattcgcctg	aaccatgaaccaagggattggacc
Acox1	ggatggtagtccggagaaca	agtctggatcgttcagaatcaag
PGC1α	ccctgccattgttaagac	Tgctgctgttcctgtttt
AFP	cccgcttccctcatcc	gaagctatcccaaactcattttcg
LCN2	tgccactccatctttcctgtt	gggagtgctggccaaataag
Prkaa2	caggccataaagtggcagtta	aaaagtctgtcggagtgctga

### Protein concentration determination by Western blot analysis

Cell pellets were resuspended in PhosphoSafe buffer (EMD Chemicals Inc. Billerica, USA), 2 mM dithiothreitol (DTT) (AppliChem GmbH, Darmstadt, Germany), 1 × Roche Complete (Roche, Mannheim, Germany), pH 7.4 supplemented with 1% 100 × Halt Protease Inhibitor Cocktail (Thermo Fisher Scientific, Darmstadt, Germany). Following sonification, lysates were centrifuged (14,000×*g*, 10 min, 4 °C) and protein concentration was determined by performing bicinchoninic acid (BCA) assay according to manufacturer’s protocol (Thermo Fisher Scientific, Waltham, Massachusetts, USA). 60 µg total protein extract were separated by SDS-PAGE and electro-blotted onto a nitrocellulose membrane (Amersham Protran, GE Healthcare Life Sciences, Freiburg, Germany). Verification of successful protein transfer was performed by Ponceau staining (0.5% in 1% acetic acid). Following 90 min incubation in 5% milk powder diluted in PBS with 0.1% Tween 20 (PBST) the membrane was incubated with the primary antibody anti-UGCG antibody (ab98030, Abcam, Cambridge, UK), anti-AuroraB/AIM1 antibody (3094, Cell Signaling Technology, Danvers, Massachusetts, USA) or anti-pan 14–3-3 antibody (sc-1657, Santa Cruz Biotechnology, Dallas, Texas, US). The IRDye680 conjugated secondary antibody (LI-COR Biosciences, Bad Homburg, Germany), was used for all proteins. Fluorescence emission and densitometric analysis was performed using the Odyssey Infrared Scanner (LI-COR Biosciences, Bad Homburg, Germany) and the Image Studio Lite software (LI-COR Biosciences, Bad Homburg, Germany). For detection of mitochondrial OXPHOS complex proteins, cells were resuspended in triton buffer (120 mM NaCl, 50 mM Tris HCl (pH 8), 1% Triton X-100) and samples incubated for 5 min at 50 °C before gel was loaded. The anti-Total OXPHOS Rodent WB Antibody Cocktail (ab110413, Abcam, Cambridge, UK) was used to label OXPHOS complexes.

### Measurement of mitochondrial respiration and glycolysis

The Seahorse XFe Analyzer (Agilent Technologies, Santa Clara, USA) was used to analyze both, oxygen consumption rate (OCR) and *extracellular acidification rate* (ECAR) in real-time. Cells were seeded and treated as described in [36]. Total cellular ATP production (J_ATPproduction_) was determined by calculating rates of glycolytic ATP production (J_ATPglc_) and oxidative ATP production (J_ATPox_) from all intracellular sources according to Mookerjee et al. and Louie et al. [27–29].

### ATP level determination

For ATP level determination the ATP Bioluminescence Assay Kit CLS II (Roche, Basel, Switzerland) was used, and samples generated according to manufacturer’s protocol. Briefly, 1 × 10^7^ cells were trypsinized, lysed and the luciferase-based assay performed.

### Substrate competition assay

Tracer assays were performed as described in Byrne et al. [6]. Briefly, a day prior to the assay 1.5 × 10^4^ cells/24-well were seeded and then incubated in Krebs Ringer Phosphate (KRP) nutrient buffer containing either D-[3–^3^H]-glucose, D-[^14^C (U)]-glucose, L[^14^C(U)]-glutamic acid or [1–^14^C]-palmitic acid. Substrate oxidation was measured capturing evolved ^14^CO_2_. For glycolysis measurements, D-[3–^3^H] glucose was separated from tritiated [^3^H]_2_O by diffusion. To quantify tracer oxidation, media was acidified and ^14^CO_2_ trapped via reaction with 0.1 ml KOH before liquid scintillation spectrometry.

### Analysis of mitochondrial DNA copy numbers per cell

To compare the nuclear to mitochondrial DNA ratio, the NovaQUANT Human Mitochondrial to Nuclear DNA Ratio Kit (Merck KGaA, Darmstadt, Germany) and SYBR select Master Mix (Thermo Fisher Scientific, Waltham, USA) were used. The qRT-PCR based kit contains primer pairs targeting two mitochondrial (ND1 and ND6) and two nuclear genes (BECN1 and NEB). By calculating ratios of the Ct values of BECN1/ND1 and NEB/ND6, the mtDNA copy number per cell was determined. DNA was isolated using KAPA Express Extract Kit (Kapa Biosystems, Wilmington, USA).

### Comparison of mitochondrial mass using NAO

Cells were seeded in 6 cm dishes and treated with 100 nM nonyl acridine orange (NAO). Cells were washed, harvested by trypsin and pelletized. Mitochondrial staining of 100.000 cells per sample was analyzed via FITC channel with the BD FACS Canto II flow cytometer and the BD FACSDiva software (BD Biosciences, Franklin Lakes, USA). Data were evaluated using FlowJo software (FlowJo LLC, Ashland, USA).

### Quantification of mitochondrial superoxide

0.5 × 10^4^ cells/96-well were seeded and incubated with 5 μM Red mitochondrial superoxide indicator for live-cell imaging (MitoSOX) (Thermo Fisher Scientific, Waltham, USA) for 30 min at 37 °C. Following a three-time washing step with PBS, fluorescence was detected by the EnSight Multimode Plate reader (PerkinElmer, Waltham, Massachusetts, US) with excitation at 510 nm and emission at 590 nm. The values are background corrected.

### Determination of total ROS levels

Total reactive oxygen species (ROS) levels determination was performed using 5-(and-6)-chloromethyl-2′,7′-dichlorodihydrofluorescein diacetate, acetyl ester (CM-H2DCFDA) (Invitrogen, Carlsbad, USA) according to manufacturer’s protocol. CM-H2DCFDA diffuses passively into cells where the acetate groups are cleaved by esterases and the thiol-reactive chloromethyl group reacts with GSH and other thiols. Subsequent oxidation leads to a fluorescent adduct, which is trapped inside the cell and is measured at Ex/Em ~ 492–495/517–527 nm.

### Determination of cell viability

To determine cell viability, a 3-(4,5-dimethylthiazol-2-yl)-2,5-diphenyltetrazolium bromide (MTT) assay was performed. 1.2 × 10^4^ cells/96-well were seeded and stimulated for 24 h with 0, 5 and 25 μM PDMP. 20 μl MTT reagent (5 mg/ml in 1 × PBS) was added, and plates incubated for three hours at 37 °C. Subsequently, media was aspirated and 80 μl MTT solvent (4 mM HCl, 0.1% NP-40, isopropanol) added. Following a 15-min incubation time, absorbance (590/620 nm) was measured on the EnSight Multimode Plate reader (PerkinElmer, Waltham, Massachusetts, US). Data are normalized to protein concentration.

### Sphingolipid level analysis of endoplasmic reticulum (ER)/mitochondria fractions

To analyze the lipid composition of the endoplasmic reticulum (ER)/mitochondria fractions, mitochondria from 1.5 × 10^7^ freshly harvested cells were isolated according to the manufacturer’s protocol using the Qproteome Mitochondria Isolation Kit (Cat. no. 37612, QIAGEN, Hilden, Germany). Sphingolipid concentrations of isolated ER/mitochondria fractions were determined by liquid chromatography tandem mass spectrometry (LC–MS/MS) as described previously [36]. Data are normalized to protein concentration.

### Isolation and identification of GEMs

Glycosphingolipid-enriched microdomains (GEMs) were isolated and identified by determination of the cholesterol level and glycosphingolipid (GSL) levels as previously described in [46]. Briefly, cells were harvested, processed and sonified in MES buffer and ultra-centrifuged in a saccharose density gradient. Eight fractions were isolated and purified from saccharose and the GEM containing fractions were identified. GlcCer and LacCer concentrations in fraction 1 to 8 were analyzed by LC–MS/MS as previously described in [46].

### Detection of intracellular-induced signaling pathways

For analysis of activated signaling pathways in NMuLi cells, the C-Series AKT Pathway Phosphorylation Array C1 (RayBiotech, Norcross, Georgia, US) was used. This antibody array detects several phosphorylated signaling proteins. The assay was performed according to the manufacturer’s protocol.

### HCC stem cell marker detection

For analysis of CD13, CD133, CD44 and CD90.1 expression, samples were acquired with a LSRII/Fortessa flow cytometer (BD Biosciences, Heidelberg, Germany) and mean fluorescence intensity (MFI) was determined using FlowJo software 7.6.1 (Treestar, Ashland, OR, USA). All antibodies and secondary reagents were titrated to determine optimal concentrations. The instrument calibration was controlled daily using Cytometer Setup and Tracking beads (BD Bioscience, Franklin Lakes, New Jersey, US). For staining of murine NMuLi UGCG OE and control cells, cell suspensions were blocked with Fc Receptor Binding Inhibitor (Miltenyi Biotec, Bergisch Gladbach, Germany) for 10 min on ice and stained with anti-CD133-PerCP-eFluor710 (eBioscience, San Diego, California, US), anti-CD44-BV510 (BD Bioscience, Franklin Lakes, New Jersey, US), anti-CD90.1-PE (eBioscience, San Diego, California, US) or anti-CD13-BV421 (BD Bioscience, Franklin Lakes, New Jersey, US) for 10 min on ice.

### Cell proliferation assays

1.2 × 10^4^ cells/24-well were seeded in media (day 0). Cells were harvested with trypsin on day 4 and cell number determined by using a Neubauer counting chamber. The second method, which was used to determine cell proliferation is the CyQUANT™ NF cell proliferation assay (Thermo Fisher Scientific, Waltham, USA). The assay was performed according to manufacturer’s protocol. Briefly, 1.2 × 10^4^ cells/96-well were seeded in control media (DMEM, phenol-red free, 1 × sodium bicarbonate solution, 1% Penicillin/Streptomycin, 10% FBS, 1.139 g/l D-glucose, 1% GlutaMAX), without glutamine supplementation or in low glucose media (0.25 g/l). On day three, growth media was removed and replaced by 100 μl/well of 1 × dye binding solution containing 1 × HBSS buffer, 1:500 CyQUANT® NF dye reagent and 1:500 CyQUANT^®^ delivery reagent. Plate was covered and incubated for 60 min at 37 °C. Subsequently, fluorescence intensity was measured at Ex/Em ~ 485/530 nm on the EnSight Multimode Plate reader (PerkinElmer, Waltham, Massachusetts, US). Data are normalized to protein concentration.

### Statistical analysis

Statistical analysis was performed with GraphPad Prism 8 software. Data are presented as mean ± standard error of the mean (SEM). Significant differences in means between two groups were assessed by unpaired *t* test with Welch’s correction and by one-way ANOVA (Tukey’s multiple comparisons test) for more than two groups. Statistical outliers were identified using a ROUT outlier test with *Q* = 1%.

## Discussion

### OXPHOS affected by UGCG

Following UGCG OE in NMuLi cells, we detected lowered basal mitochondrial respiration, lowered ATP levels and lowered cell proliferation. These changes are not ascribable to altered mitochondrial mass. Presumably, the accumulation of UGCG-derived GlcCer and LacCer in ER/mitochondria fractions induces mitochondrial superoxide and subsequently leads to the described effects in the liver cells. Our normal liver cell data are in line with studies in cancer cells showing GSL related mitochondrial dysfunction (reviewed in [35]). Although, we previously showed that in breast cancer cells, accumulation of GlcCer in ER/mitochondria fractions leads to increased mitochondrial respiration [36] and increased cell proliferation [46]. It is likely that UGCG/GlcCer have different roles in normal cells compared to cancer cells and are regulated differently. Furthermore, we detected increased dihydroceramide (dhCer) levels in ER/mitochondria fractions of UGCG OE liver cells. Interestingly, in UGCG overexpressing breast cancer cells, no changes in dhCer concentrations in ER/mitochondria fractions were detected [36]. However, dihydroceramide desaturase 1 (DEGS1) introduces a 4,5-trans-double bond into dhCer and thereby generates ceramide (Cer). Following UGCG OE in liver cells, DEGS1 mRNA expression is reduced, while the effect is not significant (data not shown). Increased DEGS1 mRNA expression might be responsible for the detected dhCer accumulation in NMuLi/UGCG OE cells. Siddique et al. showed that specifically dhCer increase impairs ATP production [39], which might be the reason for decreased ATP production following UGCG OE in NMuLi cells. No significant differences of 5,5,6,6′-tetrachloro-1,1′,3,3′ tetraethylbenzimidazoyl-carbocyanine iodide (JC-1) aggregate to monomer ratio was detected by flow cytometry, which shows that UGCG by itself does not influence mitochondrial depolarization (S2I). However, Lachkar et al. reviewed that a sphingosine kinase 2 inhibitor targets DEGS1 activity, leading to increased dhCer concentrations in prostate cancer cells. This results in decreased cell size and cell proliferation, without affecting apoptosis or autophagic cell death (reviewed in [22]). In addition, changed dhCer content results in changed membrane fluidity (reviewed in [22]). Changes of lipid composition of biological membranes affects protein activity and therefore regulates cellular processes (reviewed in [10]). Furthermore, following DEGS1 ablation, autophagy is induced in an AMPK-dependent manner [39]. This is contradictory to our data, because we detected less phosphorylated, respectively less active AMPKα. In future experiments autophagy induction analysis following UGCG OE in liver cells should be included.

### Glycolysis affected by UGCG

Decreased ATP levels in NMuLi/UGCG OE cells suggest increased adenosine monophosphate (AMP) levels. Usually, increased concentrations of AMP during mitochondrial dysfunction activate the energy sensor AMPK by binding to the γ-subunit, which leads to phosphorylation of Thr172 (reviewed in [49]). Subsequently, AMPKα is activated, which leads to induction of energy producing pathways such as glycolysis [49]. Decreased levels of phosphorylated AMPKα (P-Thr172) following UGCG OE in NMuLi cells could be the reason for lowered glycolysis in NMuLi/UGCG OE cells. mRNA expression of the catalytic subunit (α-subunit, Prkaa2) of AMPK might be increased for compensatory reasons. Interestingly, mice without hepatic expression of acetyl-CoA carboxylase (ACC) develop liver tumors [30]. ACC catalyzes the first step of de novo lipogenesis in the cytosol. Acetyl-CoA is converted into malonyl-CoA, which serves as substrate for lipid synthesis. Ishibashi et al. showed a connection between UGCG and ACC in murine fibroblasts [20]. AMPK-induced phosphorylation and subsequent inhibition of ACC leads to decreased UGCG activity and accordingly lowered GlcCer levels. Inhibition of ACC lowers the production of malonyl-CoA, which is necessary for fatty acid synthesis [19]. This arises the question whether UGCG and ACC are connected in the context of liver tumor development. We analyzed ACC1 and ACC2 mRNA and protein expression but could not detect a clear trend (data not shown). Malonyl-CoA inhibits CPT1B (rate controlling enzyme of β-oxidation), which we detected to be less expressed in NMuLi/UGCG OE cells. Reduced CPT1B indicates reduced β-oxidation, but to clearly state to what extend β-oxidation contributes to CO_2_ formation in NMuLi/UGCG OE and control cells further studies are needed. However, under various cellular stresses, induced AMPK activates AKT (P-Ser473) [18]. Interestingly, we obtained contradictory data in regards to AKT (P-Ser473) activation. AKT might be activated via mTOR (reviewed in [52]). We analyzed mRNA expression of mechanistic target of rapamycin (mTOR) and its regulating proteins regulatory-associated protein of mTOR (RPTOR) (rapamycin complex 1 (mTORC1) activity regulation) and DEP-domain containing mTOR-interacting protein (DEPTOR). mRNA levels of all three genes are not affected by UGCG OE in liver cells (data not shown), meaning AKT pathway activation seems not be induced by the mTOR signaling pathway. However, activated AKT (P-Ser473) usually phosphorylates GSK3β at serine 9, thereby causing inactivation of the protein (reviewed in [3]). Interestingly, we detected less GSK3β (P-Ser9), which means GSK3β is activated following UGCG OE in liver cells compared to control cells. Controversial data exist regarding GSK3β and its involvement in cell proliferation (reviewed in [7]). In NMuLi/UGCG OE cells, GSK3β decreases cell proliferation, which we showed with two proliferation assays. We detected slightly increased PDK1 (P-Ser241) levels following UGCG OE and PDK1 is constitutively active when phosphorylated at P-Ser241 [8]. PDK1, the master regulator of proliferative signaling, activates AKT [47], which we detected in NMuLi/UGCG OE cells by using the C-Series AKT Pathway Phosphorylation Array C1. Activation of PDK1 could be a rescue attempt of the cell to boost cell proliferation. However, UGCG inhibitor EtDO-P4 treatment clearly improves mitochondrial respiration and glycolysis of NMuLi/UGCG OE cells. Interestingly, EtDO-P4 decreases ATP production and glycolytic capacity of control liver cells. Also, EtDO-P4 increases maximal respiration and basal ECAR in NMuLi/EV-2 cells. EtDO-P4 might affect these parameters in the control cells by blocking UGCG, which is expressed at basal level in control cells. This shows the complexity of the sphingolipid metabolism and how small changes lead to profound alterations of cellular processes (reviewed in [4]). We hypothesize that control cells following EtDO-P4 treatment are forced to adapt to changed sphingolipid levels. This hypothesis is supported by our NMuLi/UGCG KD data, which show no effect of EtDO-P4 treatment on mitochondrial respiration and glycolysis. In addition, the EtDO-P4 effect is much smaller in NMuLi/EV-2 cells and NMuLi/UGCG OE cells are clearly rescued by EtDO-P4 treatment.

### Expression of tumor markers affected by UGCG

Here, we show that UGCG OE leads in liver cells to GlcCer and LacCer accumulation in GEMs and therefore changes signaling cascades in the cell. We detected reduced expression levels of EPCAM, CD13, CD133, CD90.1 and CD44, which are known liver cancer stem cell markers (LCSC) (reviewed in [44]). Furthermore, the pro-cancerous markers GLUT4 [15], GLUT6 (reviewed in [5], FGF21 [50], Xpb1 [48], PCK1 [38], Glul [26], CPT1B [1], IGF2 (reviewed in (Livingstone, 2013) and CD36 (reviewed in [43]) are lowered on mRNA levels. Notably, CD36 mRNA level is further decreased in NMuLi/UGCG KD cells compared to NMuLi/UGCG OE cells. This might be related to the very different UGCG mRNA expression in NMuLi/UGCG OE and KD cells, which affects Cer concentrations. Studies by Park et al. showed C_22:0_—C_24:0_-Cer regulate CD36 expression [31]. Interestingly, GLUT2 mRNA expression is increased following UGCG OE in NMuLi cells. It has been shown that GLUT2 [41] and Acox1 (reviewed in (Kim, 2020)) are increased in HCC, which led us to hypothesize that this might be either a compensatory effect or an early-onset of pro-cancerous processes. PGC1α has an essential role in the control of hepatic gluconeogenesis (reviewed in [24]), which indicates that PGC1α is upregulated to allow for sufficient energy supply for NMuLi/UGCG OE cells, which exhibit decreased OXPHOS and glycolysis. *Alpha fetoprotein* (AFP), together with des-γ-carboxy prothrombin (DCP) has been shown to be beneficial for serological diagnosis of HCC [12]. Since we only analyzed AFP in NMuLi cells, DCP expression must be investigated in the future. The role of LCN2 in epithelial cancers is double-edged (reviewed in [51]). Santiago-Sánchez et al. recently dicussed the role of LCN2 in a review. The majority of studies suggest LCN2 as a tumor promoter, while some studies show that increased LCN2 levels correlate with reduced tumor growth in certain cancer types (reviewed in [34]). However, it has been shown that Prkaa2 sensitizes HCC to several drugs (reviewed in Yildiz 2018*), but the precise molecular mechanisms are unknown.

### Proliferation affected by UGCG

Contradictory to our results is a study from Li et al., which showed inhibited HL-7702 (human hepatocytes) cell proliferation following UGCG (siRNA) depletion [23]. We were able to show that proliferation is impaired following UGCG OE in NMuLi cells by performing two proliferation assays. Furthermore, cell viability (MTT assay) is reduced (not statistically significant) and statistically significant increased following UGCG inhibition with PDMP in NMuLi/UGCG OE cells. Given the importance of cytokinesis for cell viability, it is interesting that we detected reduced Aurora B/AIM1 protein concentration. Aurora B/AIM1 is essential for proper cytokinesis [17]. Aurora B/AIM1 expression reaches its maximum at the G2/M cycle transition. Possibly, UGCG overexpression leads to increased degradation of Aurora B/AIM1. However, no statistically significant differences between tumor necrosis factor α (TNFα) and cytochrome C release following UGCG KD were detected by Li et al. [23]. B-cell lymphoma 2 (Bcl-2) mRNA expression is reduced, whereas, Bcl-2-associated X protein (Bax) and caspase-3 expression is induced, which indicates induction of apoptotic processes [23]. We were able to lower UGCG mRNA expression to 30% compared to control cells. In the study of Li et al., human hepatocytes were used and UGCG expression is lowered to 40%, which might be the reason for the differences in the study outcome. Also, we have not checked the effect of UGCG on apoptotic processes in normal liver cells. Unexpectedly, low glucose media conditions increase cell numbers for both NMuLi/UGCG OE and control cells. One explanation is that UGCG is less active since less glucose is available and thereby UGCG generates less GlcCer. Accordingly, UGCG/GlcCer do not induce the effects described by Li et al. [23] in NMuLi cells. This must be investigated in further studies.

## Conclusion

In this study we were able to show that UDP-glucose ceramide glucosyltransferase (UGCG) overexpression (OE) decreases mitochondrial respiration and glycolysis in normal murine liver cells. Furthermore, expression of tumor markers is lowered and UGCG-derived glucosylceramide (GlcCer) and lactosylceramide (LacCer) accumulate in glycosphingolipid-enriched microdomains (GEMs). In endoplasmic reticulum (ER)/mitochondria fractions an accumulation of GlcCer, LacCer and dihydroceramide (dhCer) was detected. This results in decreased cell proliferation (graphic abstract). Our data show that OE of UGCG per se does not induce pro-cancerous processes in normal liver cells.

### Supplementary Information

Below is the link to the electronic supplementary material.

Supplementary file1 (PPTX 942 KB)

Supplementary file2 (PPTX 75083 KB)

## Data Availability

Not applicable.

## Data availability

All data generated or analysed during this study are included in this published article (and its supplementary information files).
