# Supplementary material for: Increased glucosylceramide production leads to decreased cell energy metabolism and lowered tumor marker expression in non-cancerous liver cells
Source: Cell Mol Life Sci. 2021 Oct 9;78(21-22):7025–41. doi: 10.1007/s00018-021-03958-9 (PMC8558193; doi:10.1007/s00018-021-03958-9)
Supplement: Supplementary file 1 — Supplementary file1 (PPTX 942 KB) [file 18_2021_3958_MOESM1_ESM.pptx]

## Slide 1
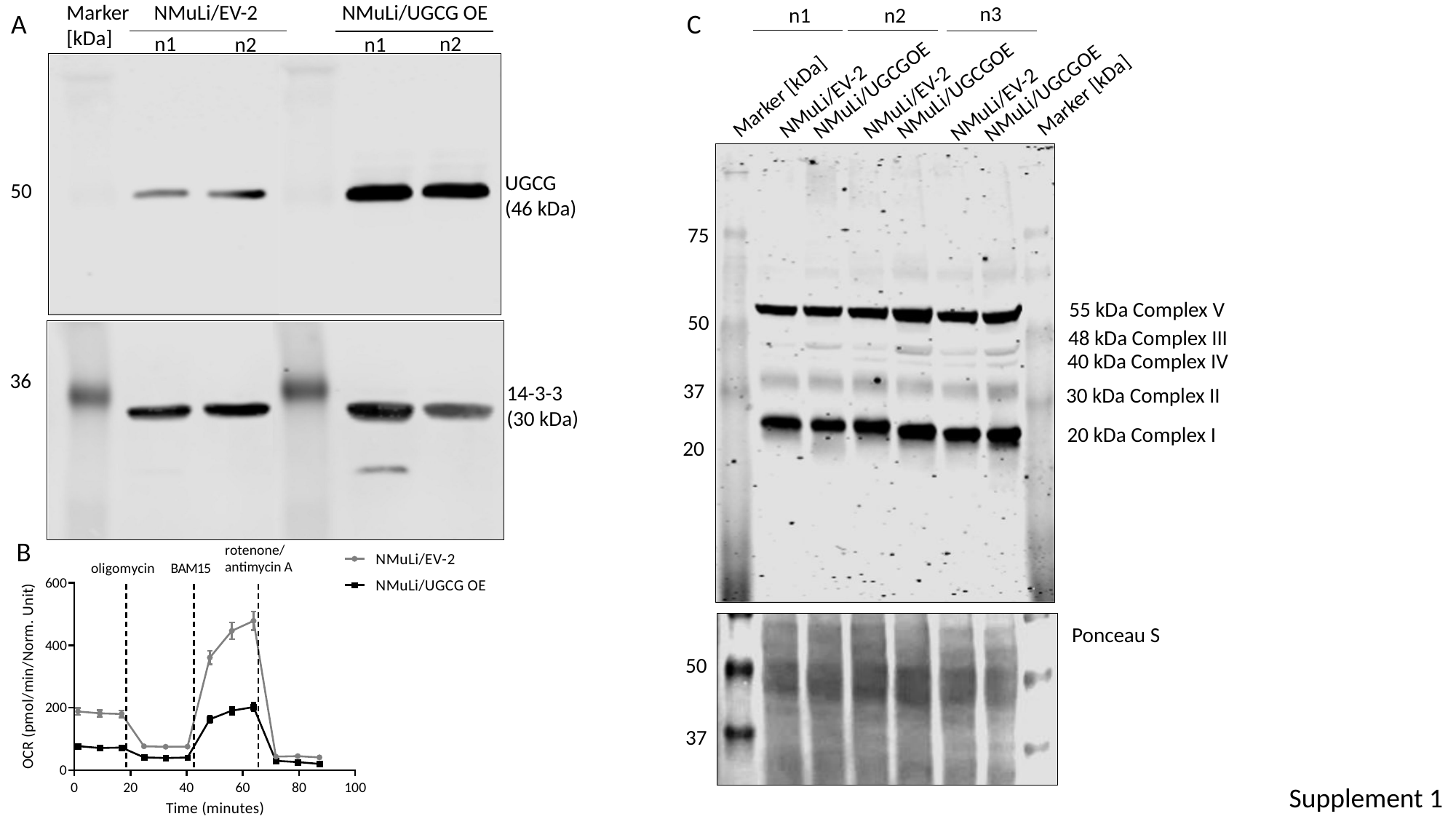

NMuLi/EV-2
NMuLi/UGCG OE
Marker
[kDa]
n3
n2
n1
A
C
n2
n1
n2
n1
NMuLi/UGCGOE
NMuLi/UGCGOE
NMuLi/UGCGOE
Marker [kDa]
Marker [kDa]
NMuLi/EV-2
NMuLi/EV-2
NMuLi/EV-2
UGCG
(46 kDa)
50
75
50
37
20
55 kDa Complex V
48 kDa Complex III
40 kDa Complex IV
30 kDa Complex II
20 kDa Complex I
36
14-3-3
(30 kDa)
B
Ponceau S
50
37
Supplement 1

## Slide 2
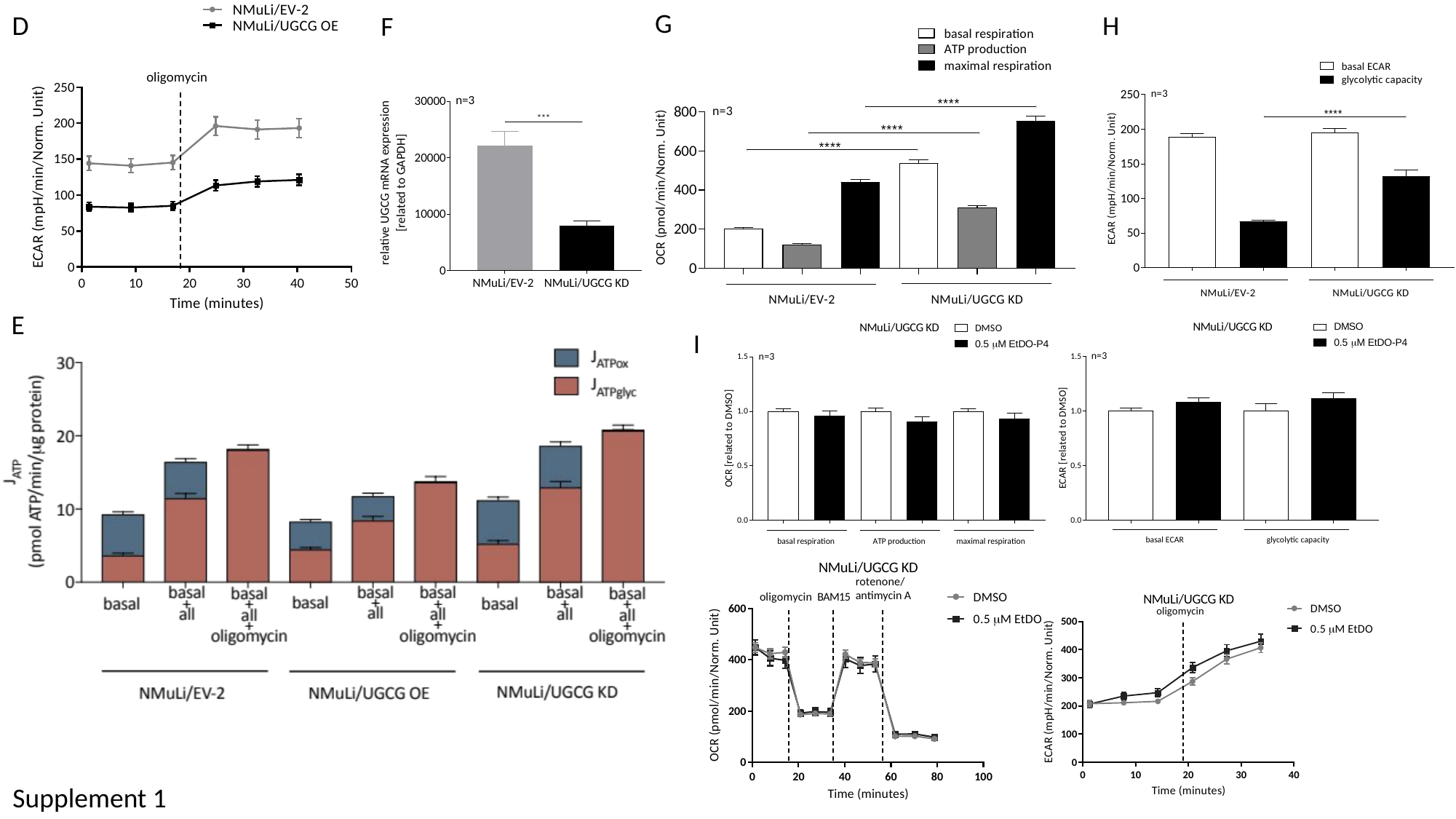

G
D
H
F
E
I
Supplement 1

## Slide 3
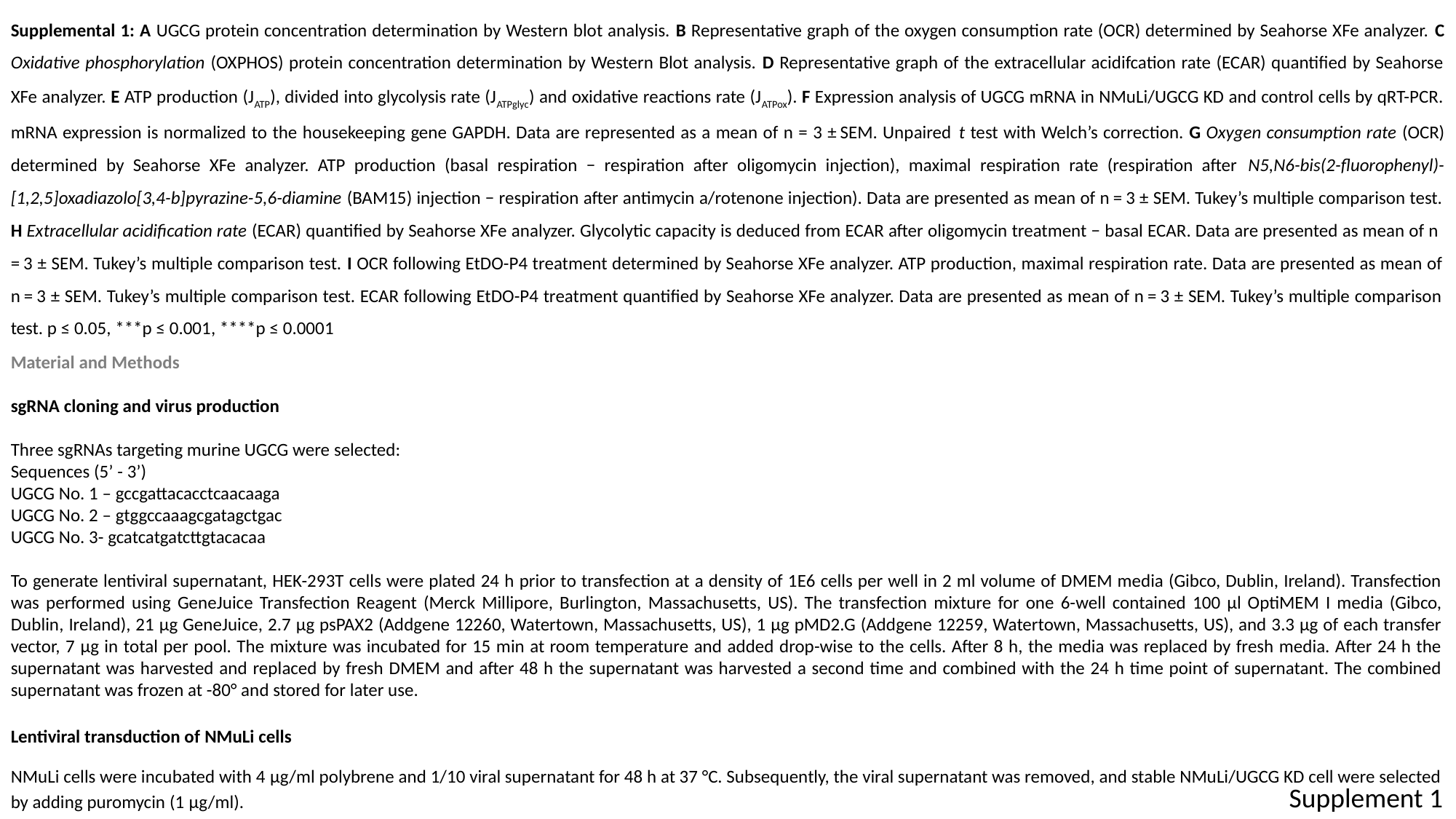

Supplemental 1: A UGCG protein concentration determination by Western blot analysis. B Representative graph of the oxygen consumption rate (OCR) determined by Seahorse XFe analyzer. C Oxidative phosphorylation (OXPHOS) protein concentration determination by Western Blot analysis. D Representative graph of the extracellular acidifcation rate (ECAR) quantified by Seahorse XFe analyzer. E ATP production (JATP), divided into glycolysis rate (JATPglyc) and oxidative reactions rate (JATPox). F Expression analysis of UGCG mRNA in NMuLi/UGCG KD and control cells by qRT-PCR. mRNA expression is normalized to the housekeeping gene GAPDH. Data are represented as a mean of n = 3 ± SEM. Unpaired t test with Welch’s correction. G Oxygen consumption rate (OCR) determined by Seahorse XFe analyzer. ATP production (basal respiration − respiration after oligomycin injection), maximal respiration rate (respiration after N5,N6-bis(2-fluorophenyl)-[1,2,5]oxadiazolo[3,4-b]pyrazine-5,6-diamine (BAM15) injection − respiration after antimycin a/rotenone injection). Data are presented as mean of n = 3 ± SEM. Tukey’s multiple comparison test. H Extracellular acidification rate (ECAR) quantified by Seahorse XFe analyzer. Glycolytic capacity is deduced from ECAR after oligomycin treatment − basal ECAR. Data are presented as mean of n = 3 ± SEM. Tukey’s multiple comparison test. I OCR following EtDO-P4 treatment determined by Seahorse XFe analyzer. ATP production, maximal respiration rate. Data are presented as mean of n = 3 ± SEM. Tukey’s multiple comparison test. ECAR following EtDO-P4 treatment quantified by Seahorse XFe analyzer. Data are presented as mean of n = 3 ± SEM. Tukey’s multiple comparison test. p ≤ 0.05, ***p ≤ 0.001, ****p ≤ 0.0001
Material and Methods
sgRNA cloning and virus production
Three sgRNAs targeting murine UGCG were selected:
Sequences (5’ - 3’)
UGCG No. 1 – gccgattacacctcaacaaga
UGCG No. 2 – gtggccaaagcgatagctgac
UGCG No. 3- gcatcatgatcttgtacacaa
To generate lentiviral supernatant, HEK-293T cells were plated 24 h prior to transfection at a density of 1E6 cells per well in 2 ml volume of DMEM media (Gibco, Dublin, Ireland). Transfection was performed using GeneJuice Transfection Reagent (Merck Millipore, Burlington, Massachusetts, US). The transfection mixture for one 6-well contained 100 µl OptiMEM I media (Gibco, Dublin, Ireland), 21 µg GeneJuice, 2.7 µg psPAX2 (Addgene 12260, Watertown, Massachusetts, US), 1 µg pMD2.G (Addgene 12259, Watertown, Massachusetts, US), and 3.3 µg of each transfer vector, 7 µg in total per pool. The mixture was incubated for 15 min at room temperature and added drop-wise to the cells. After 8 h, the media was replaced by fresh media. After 24 h the supernatant was harvested and replaced by fresh DMEM and after 48 h the supernatant was harvested a second time and combined with the 24 h time point of supernatant. The combined supernatant was frozen at -80° and stored for later use.
Lentiviral transduction of NMuLi cells
NMuLi cells were incubated with 4 µg/ml polybrene and 1/10 viral supernatant for 48 h at 37 °C. Subsequently, the viral supernatant was removed, and stable NMuLi/UGCG KD cell were selected by adding puromycin (1 µg/ml).
Supplement 1

## Slide 4
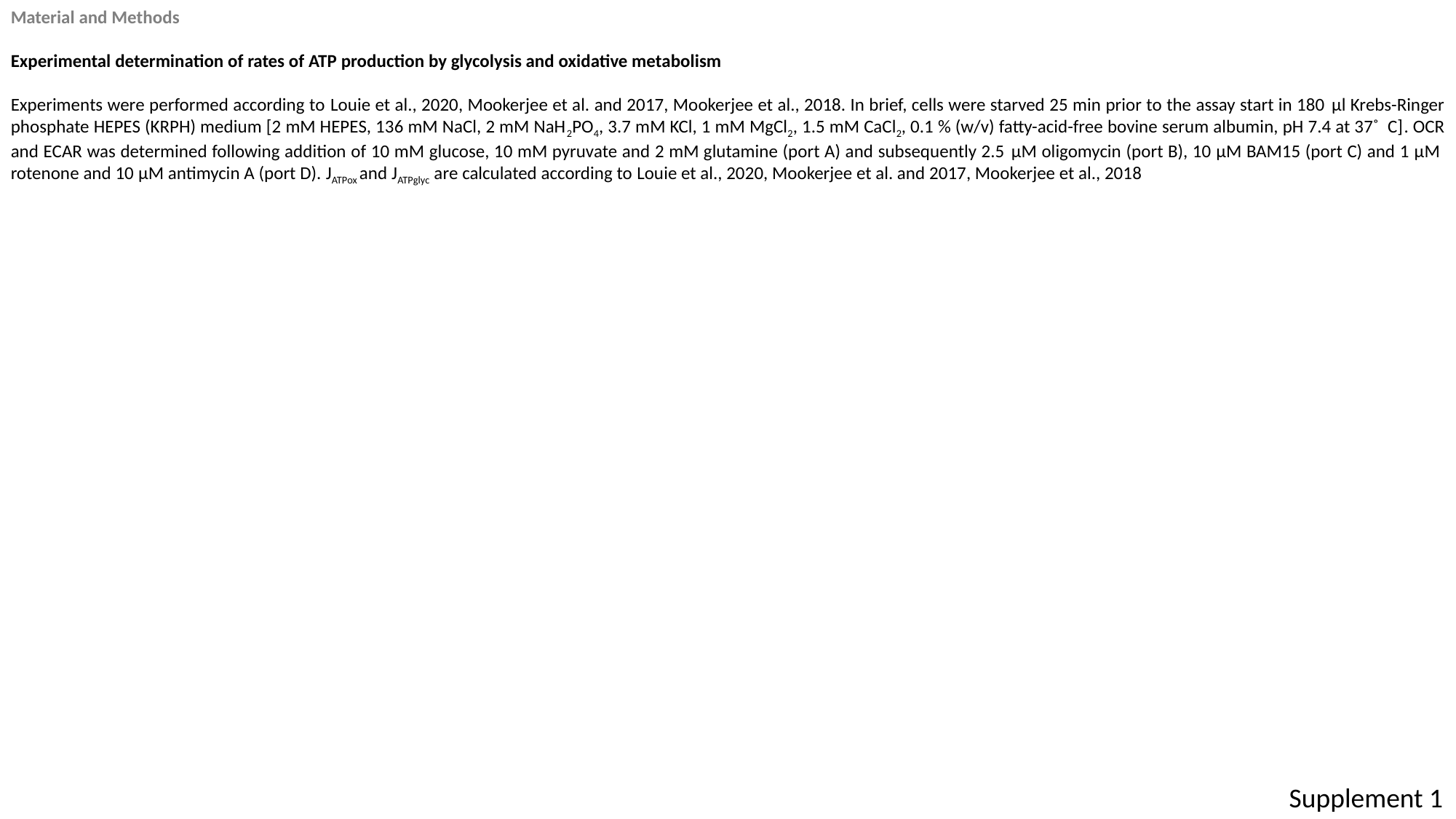

Material and Methods
Experimental determination of rates of ATP production by glycolysis and oxidative metabolism
Experiments were performed according to Louie et al., 2020, Mookerjee et al. and 2017, Mookerjee et al., 2018. In brief, cells were starved 25 min prior to the assay start in 180 μl Krebs-Ringer phosphate HEPES (KRPH) medium [2 mM HEPES, 136 mM NaCl, 2 mM NaH2PO4, 3.7 mM KCl, 1 mM MgCl2, 1.5 mM CaCl2, 0.1 % (w/v) fatty-acid-free bovine serum albumin, pH 7.4 at 37˚ C]. OCR and ECAR was determined following addition of 10 mM glucose, 10 mM pyruvate and 2 mM glutamine (port A) and subsequently 2.5 μM oligomycin (port B), 10 μM BAM15 (port C) and 1 μM rotenone and 10 μM antimycin A (port D). JATPox and JATPglyc are calculated according to Louie et al., 2020, Mookerjee et al. and 2017, Mookerjee et al., 2018
Supplement 1
