# Supplementary material for: Increased glucosylceramide production leads to decreased cell energy metabolism and lowered tumor marker expression in non-cancerous liver cells
Source: Cell Mol Life Sci. 2021 Oct 9;78(21-22):7025–41. doi: 10.1007/s00018-021-03958-9 (PMC8558193; doi:10.1007/s00018-021-03958-9)
Supplement: Supplementary file 2 — Supplementary file2 (PPTX 75083 KB) [file 18_2021_3958_MOESM2_ESM.pptx]

## Slide 1
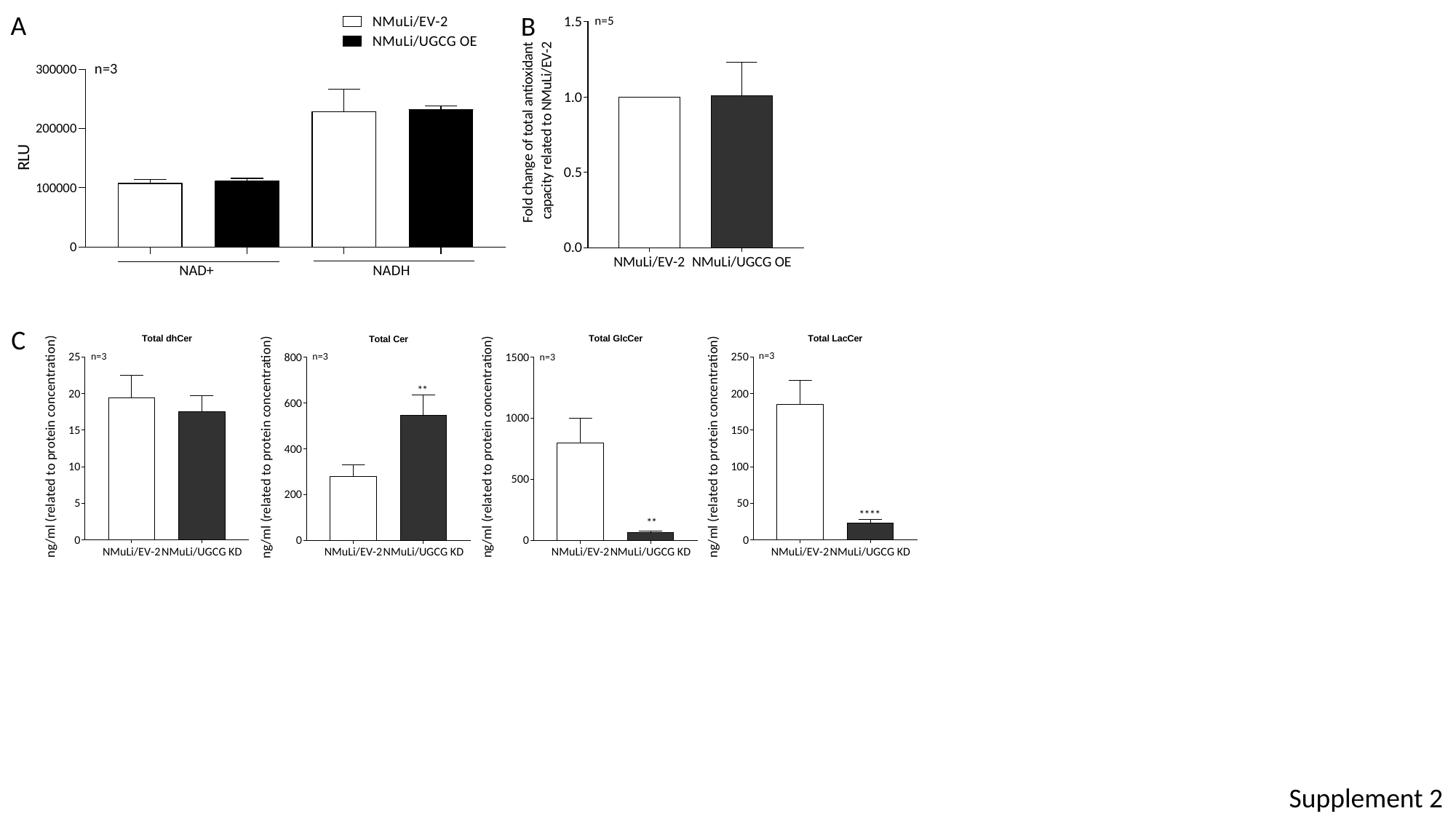

A
B
C
Supplement 2

## Slide 2
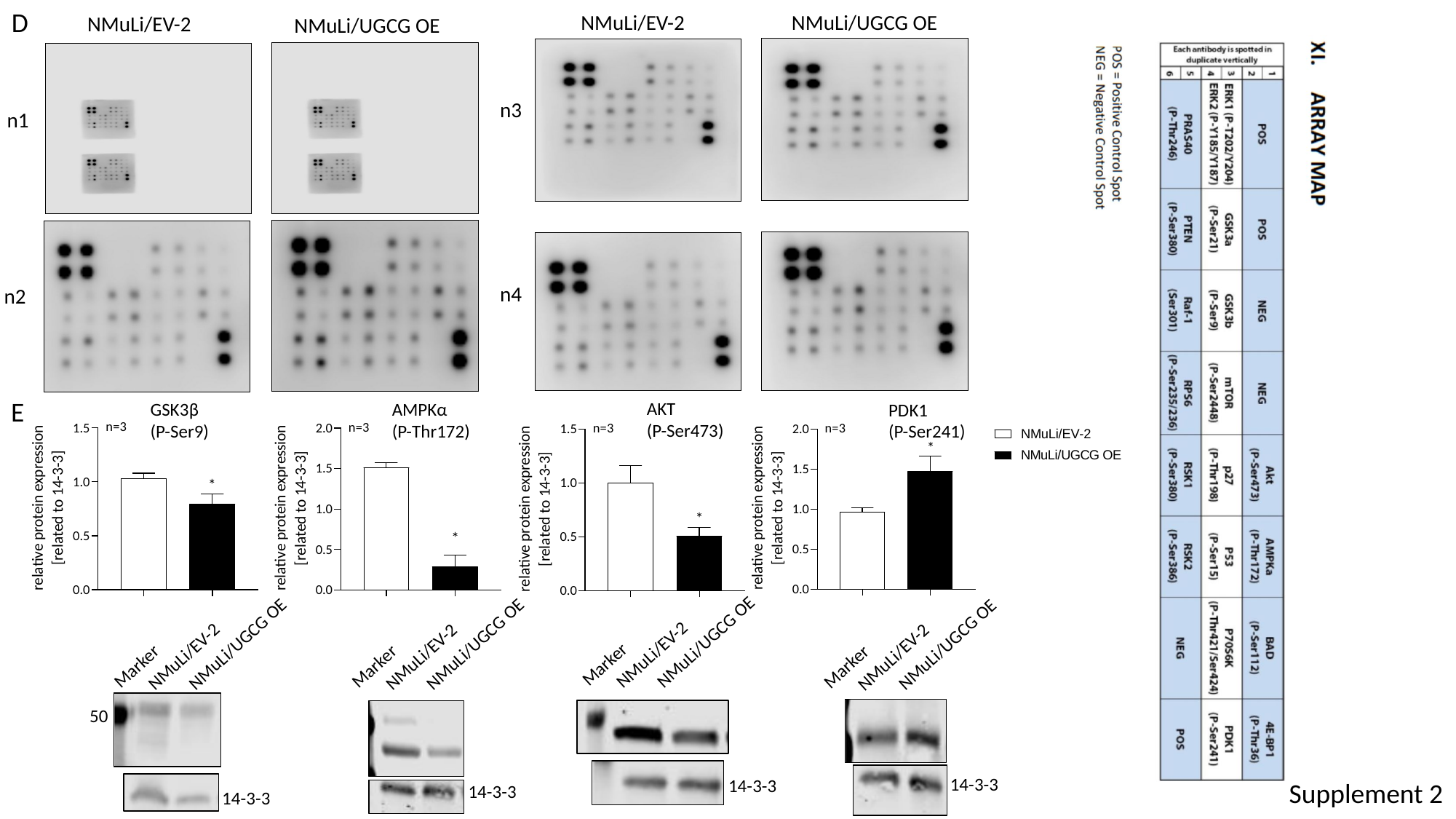

D
NMuLi/UGCG OE
NMuLi/EV-2
NMuLi/EV-2
NMuLi/UGCG OE
n3
n1
n4
n2
E
AKT
(P-Ser473)
AMPKα
(P-Thr172)
GSK3β
(P-Ser9)
PDK1
(P-Ser241)
NMuLi/UGCG OE
NMuLi/UGCG OE
NMuLi/UGCG OE
NMuLi/UGCG OE
NMuLi/EV-2
NMuLi/EV-2
NMuLi/EV-2
NMuLi/EV-2
Marker
Marker
Marker
Marker
50
14-3-3
14-3-3
Supplement 2
14-3-3
14-3-3

## Slide 3
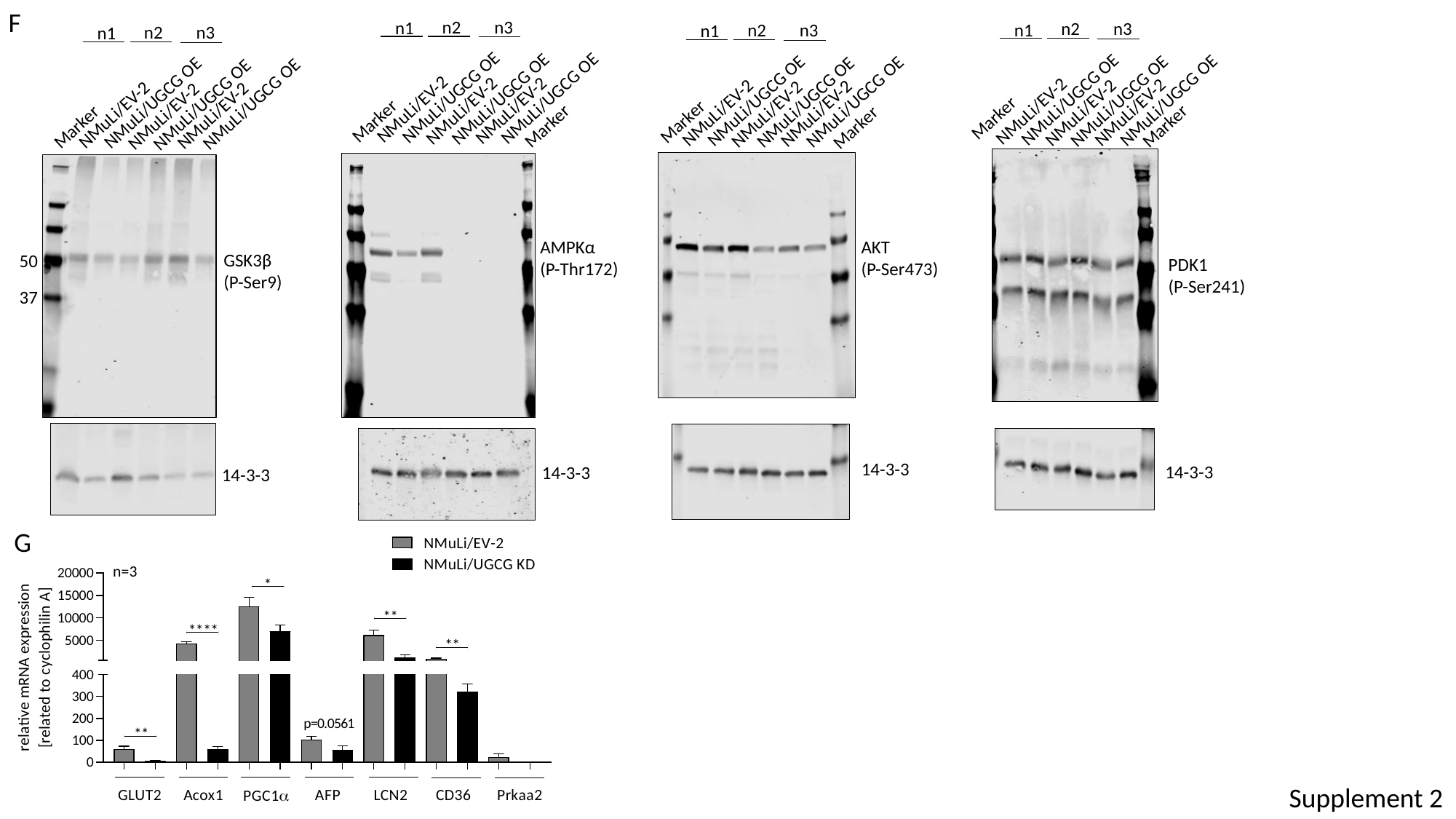

F
n2
n3
n1
n2
n3
n1
n2
n3
n1
n2
n3
n1
NMuLi/UGCG OE
NMuLi/UGCG OE
NMuLi/UGCG OE
NMuLi/UGCG OE
NMuLi/UGCG OE
NMuLi/UGCG OE
NMuLi/UGCG OE
NMuLi/UGCG OE
NMuLi/UGCG OE
NMuLi/UGCG OE
NMuLi/UGCG OE
NMuLi/UGCG OE
NMuLi/EV-2
NMuLi/EV-2
NMuLi/EV-2
NMuLi/EV-2
NMuLi/EV-2
NMuLi/EV-2
NMuLi/EV-2
NMuLi/EV-2
NMuLi/EV-2
NMuLi/EV-2
NMuLi/EV-2
NMuLi/EV-2
Marker
Marker
Marker
Marker
Marker
Marker
Marker
AKT
(P-Ser473)
AMPKα
(P-Thr172)
GSK3β
(P-Ser9)
50
PDK1
(P-Ser241)
37
14-3-3
14-3-3
14-3-3
14-3-3
G
Supplement 2

## Slide 4
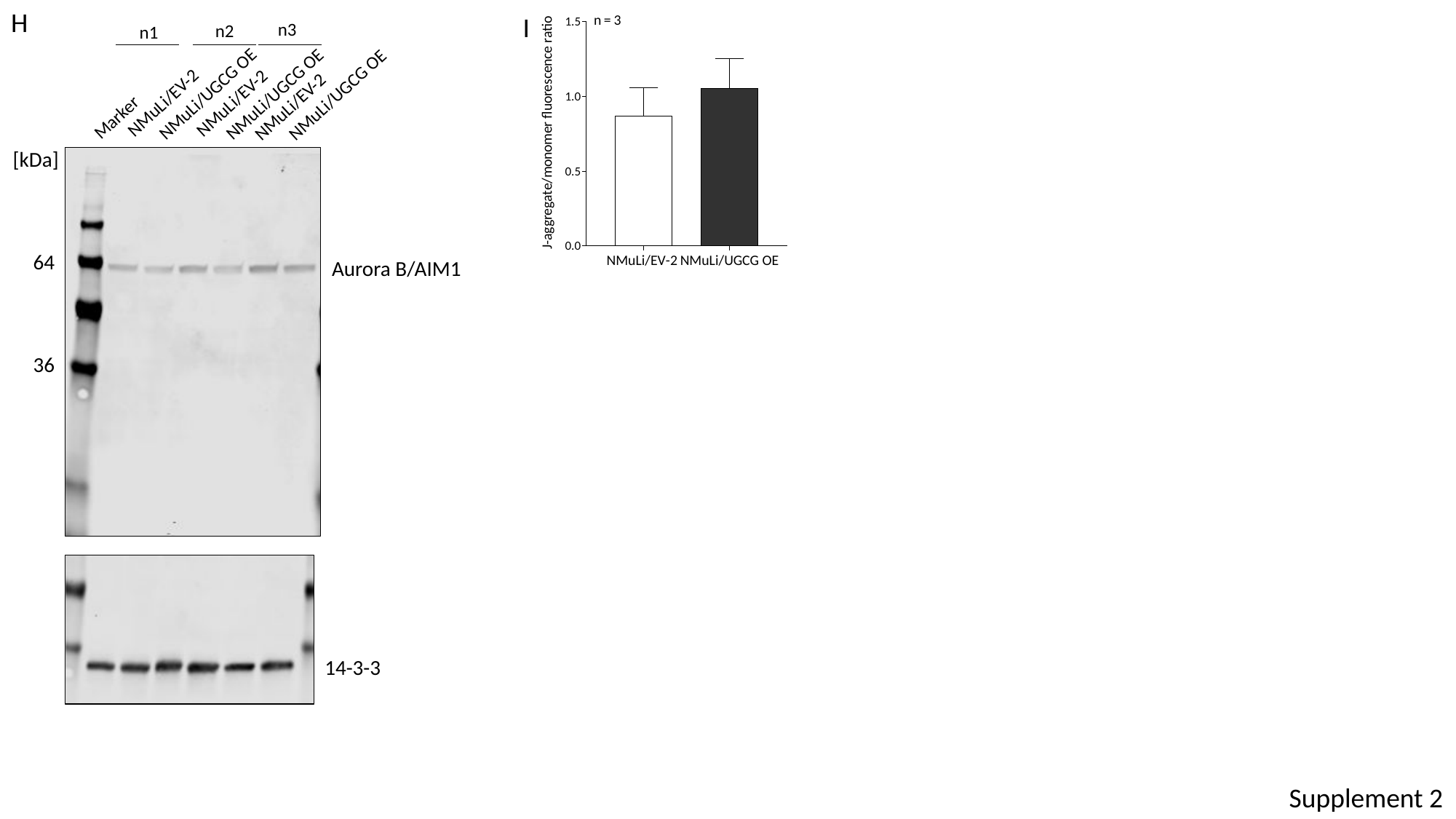

H
I
n3
n2
n1
NMuLi/UGCG OE
NMuLi/UGCG OE
NMuLi/UGCG OE
NMuLi/EV-2
NMuLi/EV-2
NMuLi/EV-2
Marker
[kDa]
64
Aurora B/AIM1
36
14-3-3
Supplement 2

## Slide 5
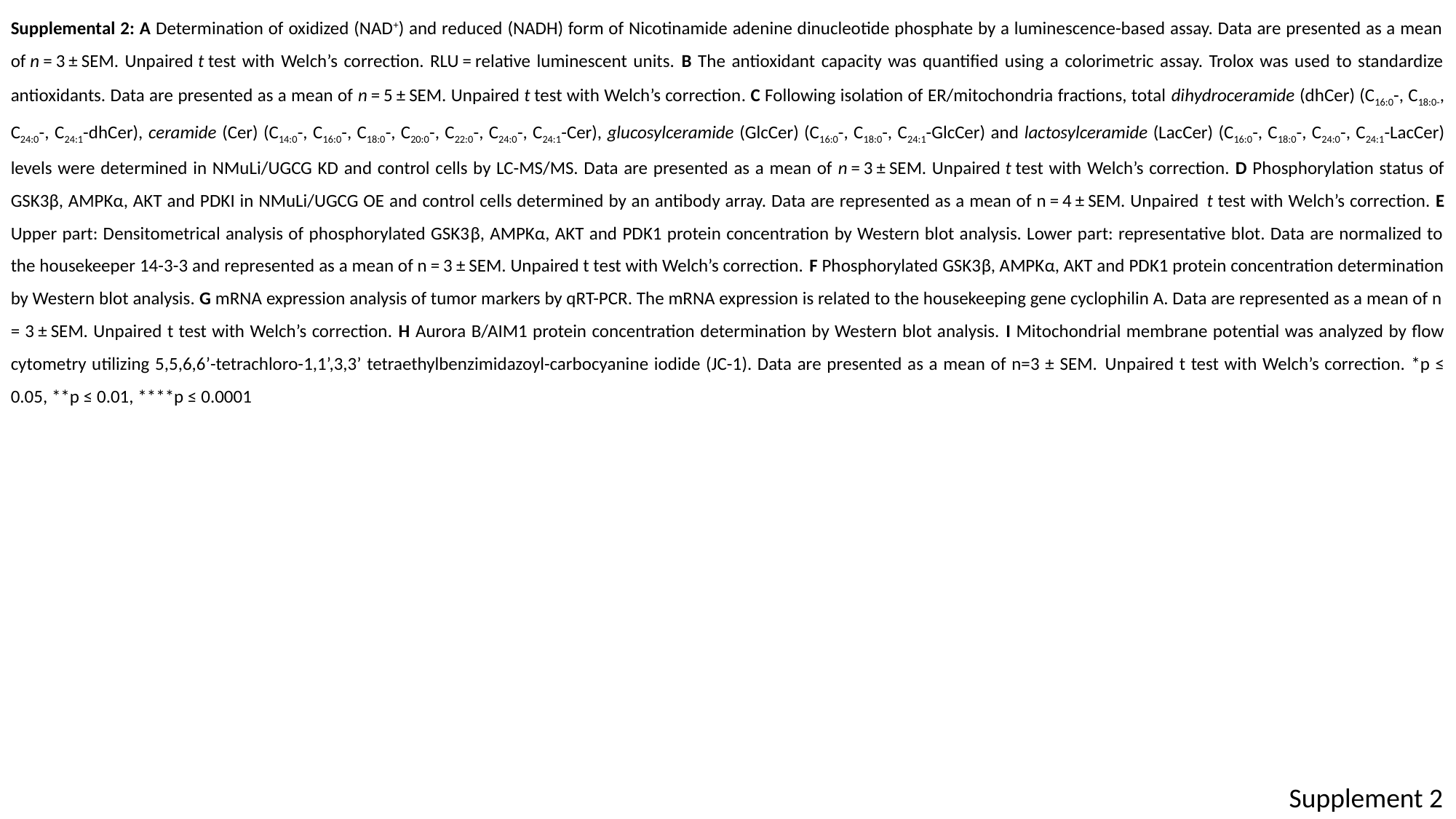

Supplemental 2: A Determination of oxidized (NAD+) and reduced (NADH) form of Nicotinamide adenine dinucleotide phosphate by a luminescence-based assay. Data are presented as a mean of n = 3 ± SEM. Unpaired t test with Welch’s correction. RLU = relative luminescent units. B The antioxidant capacity was quantified using a colorimetric assay. Trolox was used to standardize antioxidants. Data are presented as a mean of n = 5 ± SEM. Unpaired t test with Welch’s correction. C Following isolation of ER/mitochondria fractions, total dihydroceramide (dhCer) (C16:0-, C18:0-, C24:0-, C24:1-dhCer), ceramide (Cer) (C14:0-, C16:0-, C18:0-, C20:0-, C22:0-, C24:0-, C24:1-Cer), glucosylceramide (GlcCer) (C16:0-, C18:0-, C24:1-GlcCer) and lactosylceramide (LacCer) (C16:0-, C18:0-, C24:0-, C24:1-LacCer) levels were determined in NMuLi/UGCG KD and control cells by LC-MS/MS. Data are presented as a mean of n = 3 ± SEM. Unpaired t test with Welch’s correction. D Phosphorylation status of GSK3β, AMPKα, AKT and PDKI in NMuLi/UGCG OE and control cells determined by an antibody array. Data are represented as a mean of n = 4 ± SEM. Unpaired t test with Welch’s correction. E Upper part: Densitometrical analysis of phosphorylated GSK3β, AMPKα, AKT and PDK1 protein concentration by Western blot analysis. Lower part: representative blot. Data are normalized to the housekeeper 14-3-3 and represented as a mean of n = 3 ± SEM. Unpaired t test with Welch’s correction. F Phosphorylated GSK3β, AMPKα, AKT and PDK1 protein concentration determination by Western blot analysis. G mRNA expression analysis of tumor markers by qRT-PCR. The mRNA expression is related to the housekeeping gene cyclophilin A. Data are represented as a mean of n = 3 ± SEM. Unpaired t test with Welch’s correction. H Aurora B/AIM1 protein concentration determination by Western blot analysis. I Mitochondrial membrane potential was analyzed by flow cytometry utilizing 5,5,6,6’-tetrachloro-1,1’,3,3’ tetraethylbenzimidazoyl-carbocyanine iodide (JC-1). Data are presented as a mean of n=3 ± SEM. Unpaired t test with Welch’s correction. *p ≤ 0.05, **p ≤ 0.01, ****p ≤ 0.0001
Supplement 2

## Slide 6
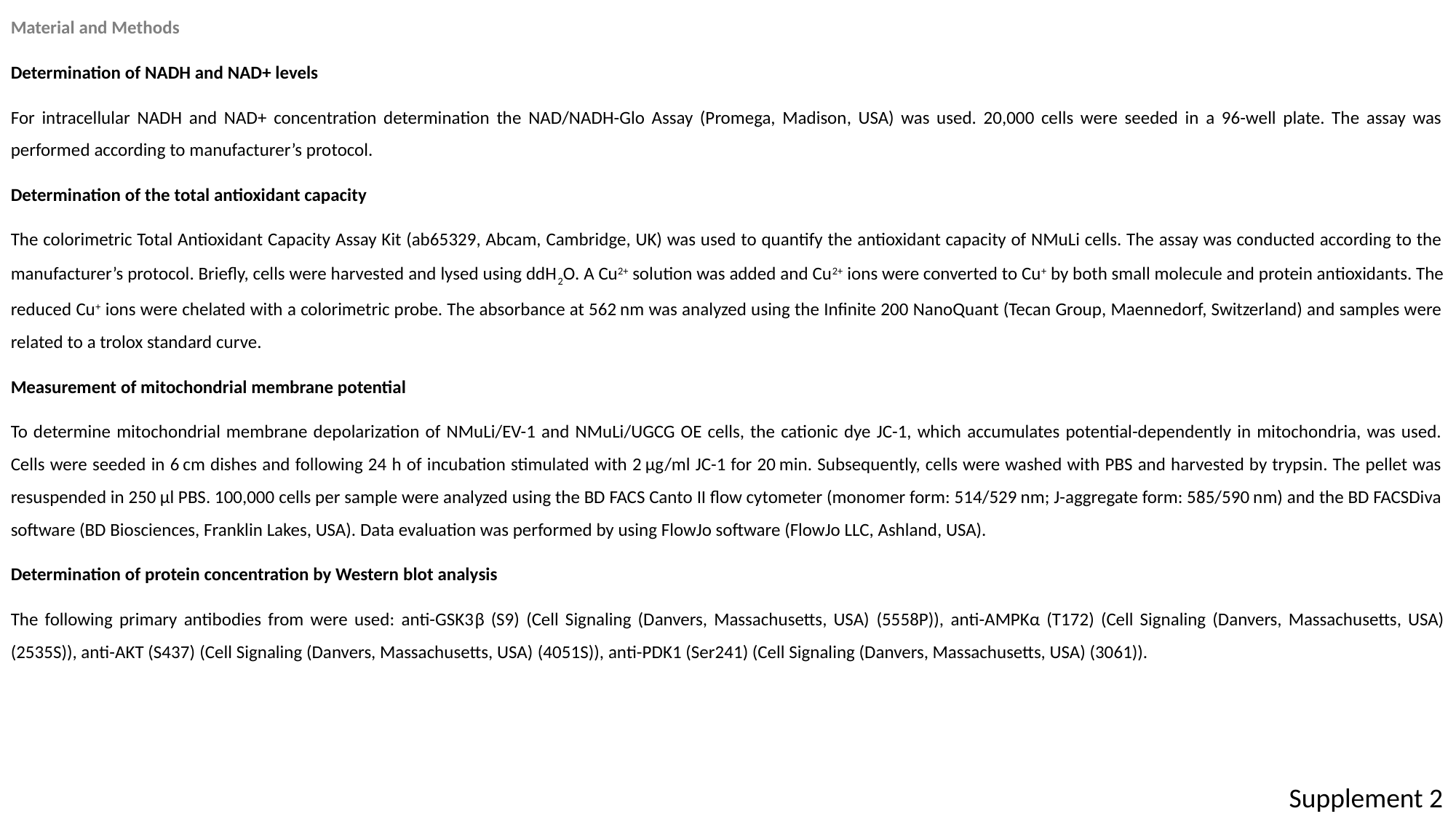

Material and Methods
Determination of NADH and NAD+ levels
For intracellular NADH and NAD+ concentration determination the NAD/NADH-Glo Assay (Promega, Madison, USA) was used. 20,000 cells were seeded in a 96-well plate. The assay was performed according to manufacturer’s protocol.
Determination of the total antioxidant capacity
The colorimetric Total Antioxidant Capacity Assay Kit (ab65329, Abcam, Cambridge, UK) was used to quantify the antioxidant capacity of NMuLi cells. The assay was conducted according to the manufacturer’s protocol. Briefly, cells were harvested and lysed using ddH2O. A Cu2+ solution was added and Cu2+ ions were converted to Cu+ by both small molecule and protein antioxidants. The reduced Cu+ ions were chelated with a colorimetric probe. The absorbance at 562 nm was analyzed using the Infinite 200 NanoQuant (Tecan Group, Maennedorf, Switzerland) and samples were related to a trolox standard curve.
Measurement of mitochondrial membrane potential
To determine mitochondrial membrane depolarization of NMuLi/EV-1 and NMuLi/UGCG OE cells, the cationic dye JC-1, which accumulates potential-dependently in mitochondria, was used. Cells were seeded in 6 cm dishes and following 24 h of incubation stimulated with 2 µg/ml JC-1 for 20 min. Subsequently, cells were washed with PBS and harvested by trypsin. The pellet was resuspended in 250 µl PBS. 100,000 cells per sample were analyzed using the BD FACS Canto II flow cytometer (monomer form: 514/529 nm; J-aggregate form: 585/590 nm) and the BD FACSDiva software (BD Biosciences, Franklin Lakes, USA). Data evaluation was performed by using FlowJo software (FlowJo LLC, Ashland, USA).
Determination of protein concentration by Western blot analysis
The following primary antibodies from were used: anti-GSK3β (S9) (Cell Signaling (Danvers, Massachusetts, USA) (5558P)), anti-AMPKα (T172) (Cell Signaling (Danvers, Massachusetts, USA) (2535S)), anti-AKT (S437) (Cell Signaling (Danvers, Massachusetts, USA) (4051S)), anti-PDK1 (Ser241) (Cell Signaling (Danvers, Massachusetts, USA) (3061)).
Supplement 2
